# Polyvalent Glycan Quantum Dots as a Multifunctional Tool for Revealing Thermodynamic, Kinetic, and Structural Details of Multivalent Lectin–Glycan Interactions

**DOI:** 10.1021/acsami.2c11111

**Published:** 2022-10-04

**Authors:** James Hooper, Yuanyuan Liu, Darshita Budhadev, Dario Fernandez Ainaga, Nicole Hondow, Dejian Zhou, Yuan Guo

**Affiliations:** † School of Food Science & Nutrition and Astbury Centre for Structural Molecular Biology, University of Leeds, Leeds LS2 9JT, United Kingdom; ‡ School of Chemistry and Astbury Centre for Structural Molecular Biology, University of Leeds, Leeds LS2 9JT, United Kingdom; § School of Chemical and Process Engineering, University of Leeds, Leeds LS2 9JT, United Kingdom

**Keywords:** multivalent lectin−glycan interaction, quantum dot, FRET, thermodynamics, kinetics, structure and function

## Abstract

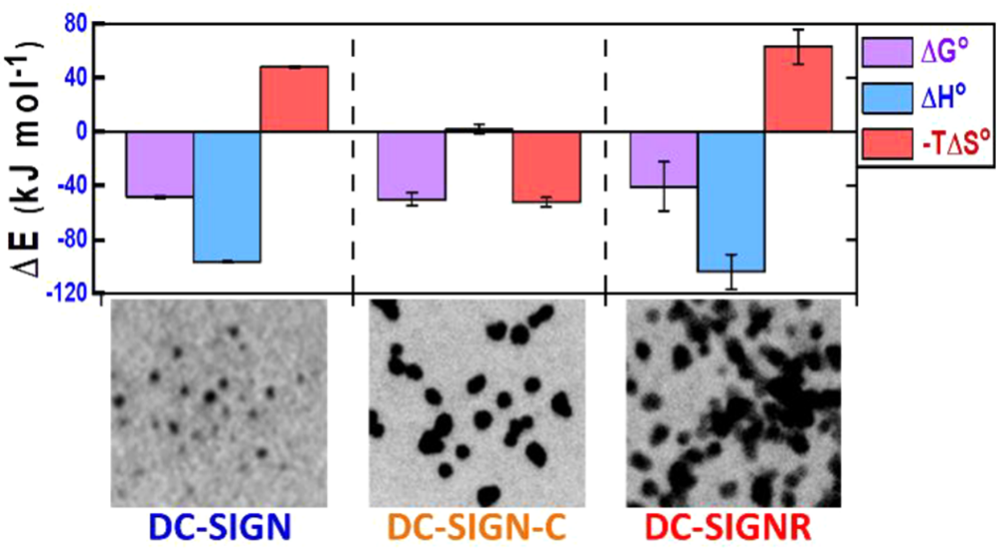

Multivalent lectin–glycan interactions (MLGIs) are widespread and vital for biology. Their binding biophysical and structural details are thus highly valuable, not only for the understanding of binding affinity and specificity mechanisms but also for guiding the design of multivalent therapeutics against specific MLGIs. However, effective techniques that can reveal all such details remain unavailable. We have recently developed polyvalent glycan quantum dots (glycan-QDs) as a new probe for MLGIs. Using a pair of closely related tetrameric viral-binding lectins, DC-SIGN and DC-SIGNR, as model examples, we have revealed and quantified their large affinity differences in glycan-QD binding are due to distinct binding modes: with simultaneous binding for DC-SIGN and cross-linking for DC-SIGNR. Herein, we further extend the capacity of the glycan-QD probes by investigating the correlation between binding mode and binding thermodynamics and kinetics and further probing a structural basis of their binding nature. We reveal that while both lectins’ binding with glycan-QDs is enthalpy driven with similar binding enthalpy changes, DC-SIGN pays a lower binding entropy penalty, resulting in a higher affinity than DC-SIGNR. We then show that DC-SIGN binding gives a single second-order *k*_on_ rate, whereas DC-SIGNR gives a rapid initial binding followed by a much slower secondary interaction. We further identify a structural element in DC-SIGN, absent in DC-SIGNR, that plays an important role in maintaining DC-SIGN’s MLGI character. Its removal switches the binding from being enthalpically to entropically driven and gives mixed binding modes containing both simultaneous and cross-linking binding behavior, without markedly affecting the overall binding affinity and kinetics

## Introduction

Lectin–glycan interactions (LGIs) are widespread and play a pivotal role in biology. As individual LGIs are intrinsically weak, and hence mostly biologically inactive, most lectins form multimeric structures, allowing them to bind multivalently with multivalent glycans to enhance affinity and form biologically relevant interactions.^1^ In the immune system, multivalent LGIs (MLGIs) are employed to recognize pathogen associated glycan patterns as a means to activate the host immune defenses against infection.^2,3^ However, undesirable nonspecific activation can lead to persistent inflammation and tissue death.^4,5^ MLGIs are also exploited by pathogens (e.g. viruses, bacteria, and fungi) to establish attachment on host cells to initiate infection or by cancer cells to suppress host immunity to assist cancer development.^6,7^ Therefore, understanding the mechanisms of MLGIs is of great importance and significance. In this regard, multivalent glycans are widely employed as research probes for MLGI mechanisms as well as potential therapeutics against specific MLGIs.^8−16^ Here, the binding mode between MLGI binding partners is critical. When MLGI binding partners have perfect spatial and orientation matches, they will form simultaneous multivalent binding and give a great affinity enhancement and hence effective intervention.^17−22^ Whereas, those that do not have such spatial/orientation matches may cross-link with each other, giving rise to a relatively low affinity enhancement and a less effective result.^19−21^ However, information regarding the majority of MLGI binding modes and how different binding modes affect the affinity and underlying binding thermodynamics and kinetics remains largely unexplored. This is presumably due to limitations of current biophysical techniques in probing such complex and flexible interactions. For example, isothermal titration calorimetry (ITC),^23,24^ and surface plasmon resonance (SPR)^25^ are two of the most widely employed techniques to study the thermodynamics and kinetics of binding interactions, including MLGIs. However, ITC cannot accurately determine the affinity of very strong interactions (e.g., with equilibrium binding dissociation constants, *K*_d_s, at the low nanomolar level or below).^26,27^ Moreover, cross-linking can make it difficult to interpret the ITC data.^28^ It is also difficult to dissect how individual LGIs contribute and control the overall MLGI affinity and specificity by SPR because these are strongly affected by the density and orientation of the surface immobilized binding partner.^29^ In addition, SPR measures the binding interactions occurring at the surface–solution interface, a very different environment from that in solution. Hence, the kinetic data measured by SPR may not be directly transferrable to that in solution. Thus, these conventional biophysical techniques can only provide some, but not a whole set, of key biophysical parameters (e.g., binding thermodynamics, kinetics, binding modes, and binding site orientation), which are important for both the fundamental understanding and therapeutic development against specific MLGIs.

Meanwhile, over the past two decades, the strongly fluorescent quantum dots (QDs) have emerged as a powerful probe for biological and biomedical research. In particular, the QDs strong and robust fluorescence has been widely exploited as sensitive QD-FRET (Förster resonance energy transfer) readouts in broad biosensing, bioanalytical and diagnostic assays, and bioimaging applications.^30−36^ Compared to other readout strategies, the QD-FRET has the advantages of high sensitivity, simple, separation-free detection, and excellent assay robustness because of its ratiometric character. In this regard, we have recently demonstrated that densely glycosylated QDs (glycan-QDs) are powerful new probes for MLGIs. We have shown that glycan-QDs can not only provide quantitative MLGI binding affinities via the QD-FRET readout^19,20,37^ but also dissect their exact binding modes by S/TEM imaging of binding-induced QD assemblies.^20^ Using the tetrameric DC-SIGN and DC-SIGNR (collectively denoted as DC-SIGN/R, hereafter) as model lectins, we have found that despite sharing ∼80% amino acid identify, an overall tetrameric architecture with identical monovalent mannose binding motifs,^38,39^ their binding properties with mannose-α-1,2-mannose (DiMan)-capped QDs (QD-DiMan) are very different, where DC-SIGN binds strongly via simultaneous binding with one QD and DC-SIGNR binds more weakly via cross-linking with multiple QDs.^20^ We have revealed that QD-DiMan only potently blocks DC-SIGN-mediated, but not DC-SIGNR-mediated, virus infections, and their potencies are positively linked to lectin binding affinities.^20^ We have attributed such differences to a subtle orientation difference of their four carbohydrate recognition domains (CRDs), while all four CRDs point uprightly in DC-SIGN; those in DC-SIGNR are split into two pairs and point sideways.^19,20^ This CRD orientation difference may account for their distinct virus transmitting properties. For instance, DC-SIGN was found to be more effective in transmitting some HIV strains than DC-SIGNR,^40^ while only DC-SIGNR, but not DC-SIGN, could effectively transmit West Nile Virus for infection.^41^

The close structural similarity and monovalent mannose specificity, yet distinct multivalent binding mode with QD-DiMan, make DC-SIGN/R a perfect pair of model lectins to study how binding modes affect binding thermodynamics and kinetics of MLGIs as well as their structural bases behind MLGI specificity. Moreover, DC-SIGN/R plays a key role in facilitating the infection of a wide range of viruses, e.g., HIV, HCV, Ebola, Zika, and more recently SARS-CoV-2;^40−44^ their MLGI biophysical details are thus of great biomedical importance and significance. These are not only for understanding their basic structural and biophysical mechanisms but also for guiding the design of multivalent glycan entry inhibitors for blocking DC-SIGN/R-medicated viral infections. This antiviral mode can avoid virus mutation and develop resistance and thus can be advantageous over other antiviral strategies.^8,9,13−15^ In addition, DC-SIGN targeting multivalent glycans can be harnessed as potential new therapeutics against cancer, allergy, and other immune dysregulation diseases, by exploiting DC-SIGN’s powerful immune regulation functions.^2−7^

In this paper, we have significantly extended the capability of the QD-DiMan probes for MLGIs by studying their binding thermodynamics via measuring temperature-dependent binding affinities in combination with Van’t Hoff analysis. We have also studied their binding kinetics via stopped flow fluorescence. Additionally, we have identified that a 16 amino acid segment located at the C-terminus of DC-SIGN, which is absent in DC-SIGNR and plays an important role in DC-SIGN’s ability in HIV transmission,^40^ is critical in defining DC-SIGN’s binding thermodynamics and binding mode. This work thus provides a significant development in establishing glycan-QDs as a powerful new platform for studying MLGIs, extending their capability for probing a range of biophysical parameters, mechanisms, and protein structure–function relationships.

## Results and Discussion

### Materials Synthesis and Characterization

**Figure 1:**
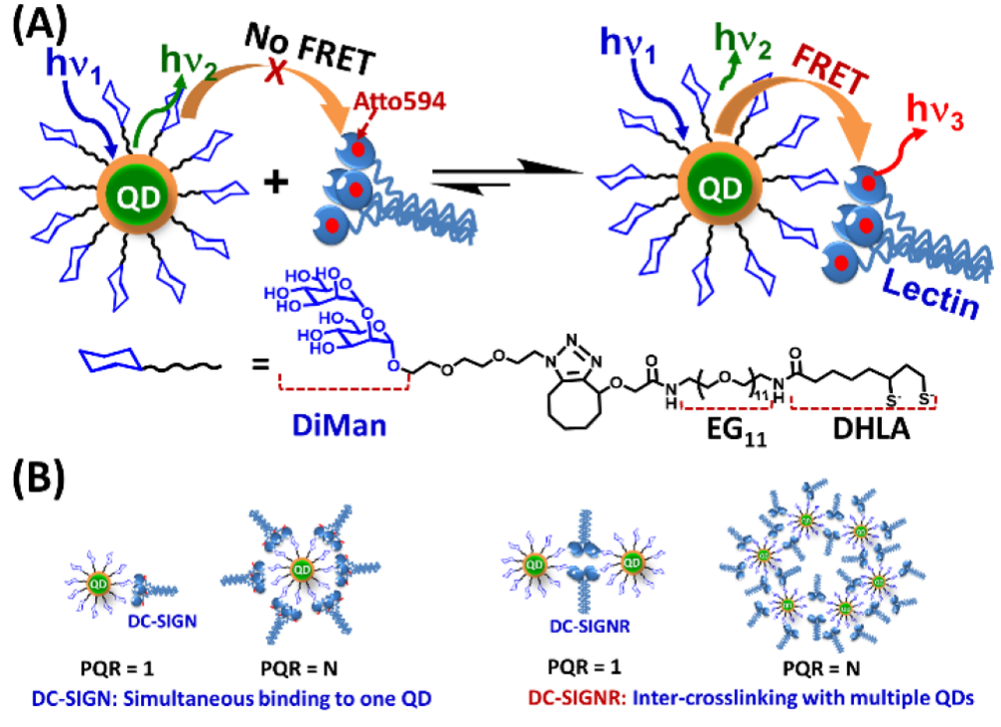
(A) Schematic showing the QD-FRET readout for QD-DiMan–lectin (dye labeled) affinity measurement. Only lectins bound to QD-DiMan, but not those unbound, produce a QD-sensitized dye FRET signal upon exciting the QD. The FRET signal is directly correlated to the binding/dissociation equilibrium between QD-DiMan and labeled lectin. The chemical structure of DHLA-EG_11_-DiMan ligand is shown beneath. (B) Schematic showing the different binding modes for DC-SIGN/R leading to different QD assemblies. The simultaneous DC-SIGN-QD binding leads to individual QD particles at high protein:QD ratios (PQRs), whereas the cross-linking between DC-SIGNR and QD results in a number of QDs being assembled together as large-scale assemblies.

A dihydrolipoic acid–undeca(ethylene glycol)-mannose-α-1,2-mannose (DHLA-EG_11_-DiMan)-based multifunctional glycan ligand (see Figure 1 for its chemical structure) was synthesized by using our previous procedures.^20^ Additionally, a DHLA–tri(ethylene glycol)-based ligand terminated with a di(ethylene glycol) group (denoted as DHLA-EG_3_-OH) was also synthesized as a negative control (see Figure S1 for its chemical structure). Each glycan ligand contains three functional domains: a DHLA group for strong QD anchoring via chelative zinc thiolate coordination;^31^ a flexible EG_11_ linker for imposing high water solubility, excellent stability, and resistance against nonspecific adsorption;^45,46^ and a terminal DiMan group for specific DC-SIGN/R binding.^20^

A CdSe/ZnSe/ZnS core/shell/shell QD (λ_EM_ ∼ 550 nm, core diameter ∼3.9 nm, quantum yield = 62%) was employed to construct the glycan-QDs. It also acted as the donor for developing the QD-FRET-based binding assays. Cap exchange using deprotonated DHLA-EG_11_-DiMan in a homogeneous solution was employed to make DHLA-EG_11_-DiMan-capped QD (denoted as QD-DiMan, hereafter) as reported previously.^19,20^ A QD capped with the DHLA-EG_3_-OH control ligand (denoted as QD-OH hereafter) was also prepared as a negative control for lectin binding. Both QDs were found to be monodisperse, relatively compact (with hydrodynamic diameters, *D*_h_, of ∼12 and ∼9 nm for QD-DiMan and QD-OH, respectively; see Figure S2), and highly stable. No changes of physical appearance or precipitation were observed after storage for 1 month in a fridge. The average glycan valency per QD was estimated as 212 ± 69 by measuring the difference between the amount of ligand added and that remaining unbound post-cap-exchange (Figure S3).^20^ By calculating the average deflection angle of the glycan ligands, and using the *D*_h_ value above, the average inter-glycan distance was estimated to be 1.7 ± 0.3 nm (Table S1).^36^

The soluble extracellular segment of DC-SIGN/R, which has shown to faithfully retain the tetrameric structure and glycan binding properties of full length proteins, were used in all glycan-QD binding studies.^19,20^ DC-SIGN with its C-terminal 16 amino acids truncated (denoted as DC-SIGN-C hereafter) was constructed using standard molecular biology techniques, and the construct was confirmed by DNA sequencing. All labeled proteins were expressed, purified, and labeled with maleimide-modified Atto-594 dye (λ_EM_ ∼ 628 nm), which acted as the FRET acceptor, via a site-specific cysteine mutation at Q274 in DC-SIGN and DC-SIGN-C or R287 in DC-SIGNR, as before.^19,20^ The labeling positions lie outside of the lectins’ glycan binding pockets; hence, Atto-594 labeling does not affect their glycan binding properties as confirmed previously.^20^ The QD-Atto-594 FRET pair has good spectral overlap with a respectable Förster radius (*R*_0_ ∼ 5.7 nm, Figure S4), ensuring that efficient FRET can occur. Moreover, there is little overlap between the QD and dye emission spectra, making it easy to differentiate donor and acceptor fluorescence without the need of spectral deconvolution.^20^ The proteins were characterized by high-resolution mass spectrometry (HRMS), UV–vis spectroscopy, and dynamic light scattering to confirm their identity and size (Figures S5–S8). All three lectins were found to form stable tetramers with comparable hydrodynamic diameters (*D*_h_s) of ∼14 nm (Figure S6). The average dye labeling efficiency was ∼85% per monomer for DC-SIGN/R and ∼75% for DC-SIGN-C (Figure S7).

### Quantifying Binding Affinity and Thermodynamics via QD-FRET

The principle of the QD-FRET readout for quantifying the DC-SIGN/R (Atto-594 labeled) binding with QD-DiMan is shown schematically in Figure 1. Because FRET can only happen over a short distance (e.g., <10 nm), any unbound lectins (acceptors) would be too far away to participate FRET interactions with the QD donor and hence will not contribute to the FRET signal. Thus, the observed FRET signal is directly linked to the equilibrium of QD-DiMan–lectin binding and, more specifically, the amount of lectins bound to the QD. This is a distinct advantage of the QD-FRET readout, allowing for binding assays to be performed in homogeneous solutions without separation.^34,35,47^ The apparent binding equilibrium dissociation constants, *K*_d_s (the inverse of the equilibrium association constant, *K*_a_, i.e., *K*_d_ = 1/*K*_a_), between QD-DiMan and the lectins were measured via our recently established method.^20^ Briefly, the fluorescence spectra of premixed QD-DiMan + lectin samples with varying protein concentration, but under a fixed protein:QD molar ratio (PQR) of 1:1 for DC-SIGN or 10:1 for DC-SIGNR, were recorded at a fixed excitation wavelength (λ_ex_) of 450 nm. This λ_ex_ corresponds to the absorption minimum of the Atto-594 receptor, thereby minimizing the dye direct excitation background. A higher PQR for DC-SIGNR was used to compensate for its relatively low FRET signal due to weak binding.^20^ Note that we have used *K*_d_ rather than *K*_a_ to describe all QD–lectin binding studies because *K*_d_ is a more straightforward and widely used indicator of binding strength than *K*_a_, especially for those involving biological binding partners. It also indicates a binder concentration that yields 50% binding (and 50% dissociation).

**Figure 2:**
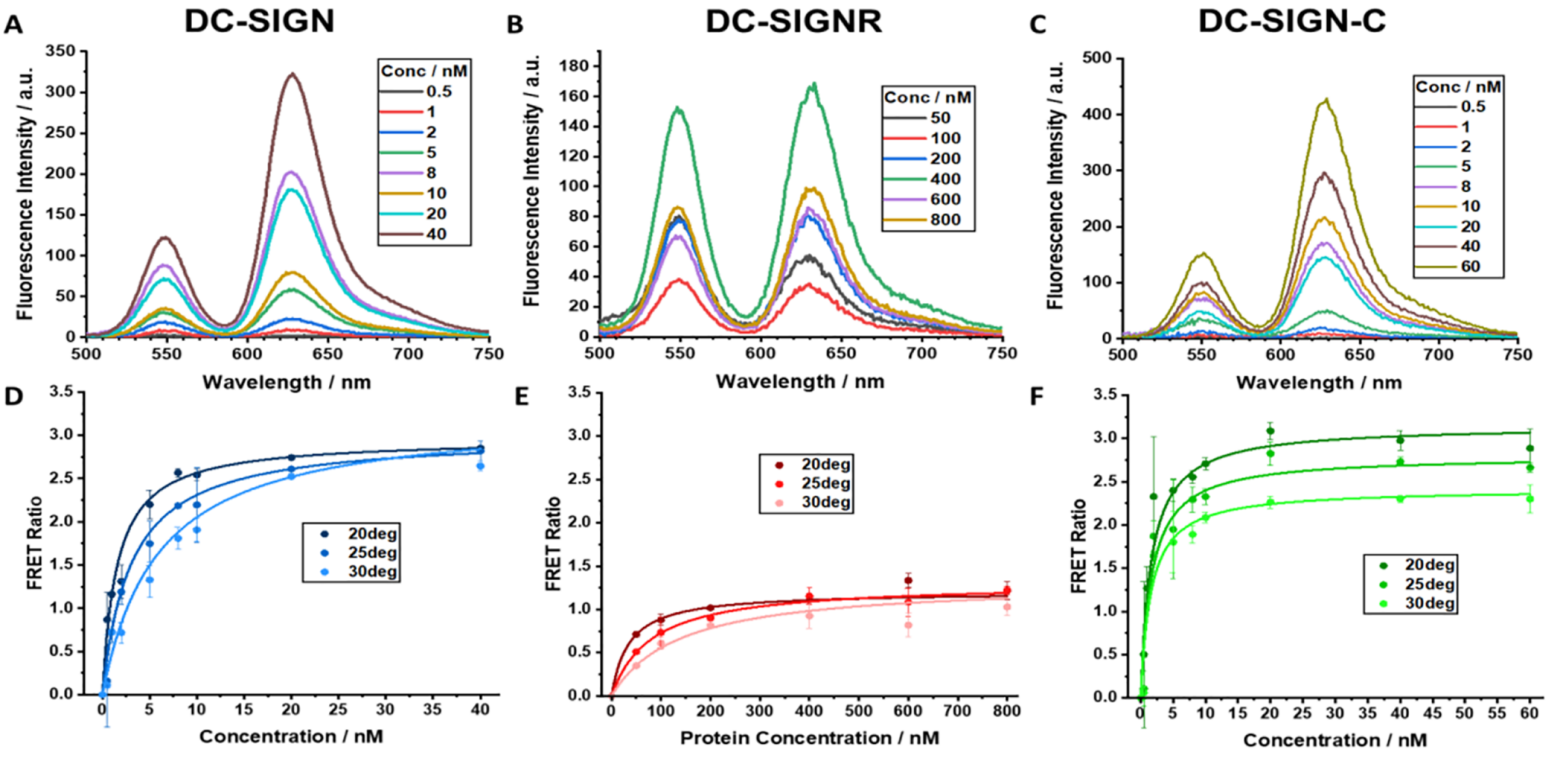
Background-corrected fluorescence spectra of different concentrations of a mixture of QD-DiMan with Atto-594 labeled lectins for (A) a 1:1 ratio of QD:DC-SIGN, (B) a 1:10 ratio of QD:DC-SIGNR, and (C) a 1:1 ratio of QD:DC-SIGN-C. The corresponding apparent FRET ratio–protein concentration relationships at three different temperatures, fitted by eq 1, for (D) 1:1 mixed QD and DC-SIGN, (E) 1:10 mixed QD and DC-SIGNR, and (F) 1:1 mixed QD and DC-SIGN-C. Error bars represent the standard deviations (SDs) of triplicate experiments at each concentration.

The corresponding dye direct excitation background corrected fluorescence spectra (Figure 2A,B) revealed that while both the fluorescence intensities of the QD donor (*I*_D_, at ∼550 nm) and Atto-594 acceptor (*I*_A_, at ∼628 nm) were increased with increasing concentration, *I*_A_ increased more quickly than *I*_D_, leading an increasing apparent FRET ratio (*I*_A_/*I*_D_) at higher concentrations before reaching saturation (Figure 2D,E). In contrast, incubating the labeled lectins with the QD-OH control without the terminal glycan did not produce any noticeable FRET signals, confirming that the FRET signal observed here was due to specific lectin–glycan interactions (Figure S9). Neither the QD nor the labeled proteins exhibited significant absorption at λ_ex_ of 450 nm to affect the FRET measurement via inner filter effect. Their absorbance at 450 nm were <0.01 even at the highest concentration, e.g., 80 nM for the QD and 800 nM for protein, and their fluorescence intensity–concentration relationships were both perfectly linear across the range of concentrations studied (Figure S10).

The apparent FRET ratio–concentration relationships were fitted with the Hill’s equation (eq 1) to derive the apparent binding *K*_d_ values (Figure 2D,E and Table 1),^20^ where *x* is the protein concentration, *F* is the apparent FRET ratio, *F*_max_ is the maximal FRET ratio at saturated binding, and *n* is the Hill coefficient. Here, *n* = 1 was assumed for all fittings. As most affinity assays were performed under a PQR of 1, most QDs should be bound with just one lectin; thus, no intermolecular lectin–lectin interactions were expected to inhibit or promote the lectin–QD binding.^48^
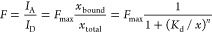
1

**Table 1:** Fitting Parameters of the FRET Curves for QD-DiMan Binding with Labeled DC-SIGN, DC-SIGN-C, and DC-SIGNR at Varying Temperatures (*R*^2^ > 0.99 for All Fits, SDs Represent Fitting Errors)

protein	*T*/°C	*K*_d_/(10^–9^ M)	*F*_max_
DC-SIGN	20	1.54 ± 0.07	3.0 ± 0.1
	25	3.00 ± 0.04	3.00 ± 0.01
	30	5.9 ± 1.7	3.3 ± 0.2
DC-SIGN-C	20	1.62 ± 0.28	3.15 ± 0.08
	25	1.67 ± 0.48	2.80 ± 0.07
	30	1.56 ± 0.50	2.42 ± 0.12
DC-SIGNR	20	35 ± 2	1.20 ± 0.02
	25	80 ± 6	1.31 ± 0.04
	30	130 ± 10	1.30 ± 0.09

Previously, most QD-FRET binding assays were performed by varying the amount of protein (or other binder) while maintaining a fixed QD concentration to obtain the fluorescence–concentration relationships from which apparent *K*_d_ values were derived.^49^ While such a method can provide accurate *K*_d_ values for weak binders (e.g., true *K*_d_ ≫ 50% of the QDs concentration at maximal binding capacity, i.e., *C*_QD_ × *N* × 50%, where *C*_QD_ and *N* are the QD concentration and the maximum number of proteins that can bind to each QD, respectively), it cannot provide accurate measurement for strong binders (e.g., true *K*_d_ < *C*_QD_ × *N* × 50%), where the measured *K*_d_ values will simply equal *C*_QD_ × *N* × 50%. In contrast, our above method does not have such limitations and can provide robust *K*_d_ measurements for both strong and weak binding partners. This is because the *I*_A_/*I*_D_ ratio is linearly proportional to the amount (or fraction, under a fixed PQR) of lectins bound to the QD in the binding system, and thus it is independent of the protein concentration or QDs binding capacity, making it a highly robust parameter for *K*_d_ quantification.^20,37^

**Figure 3:**
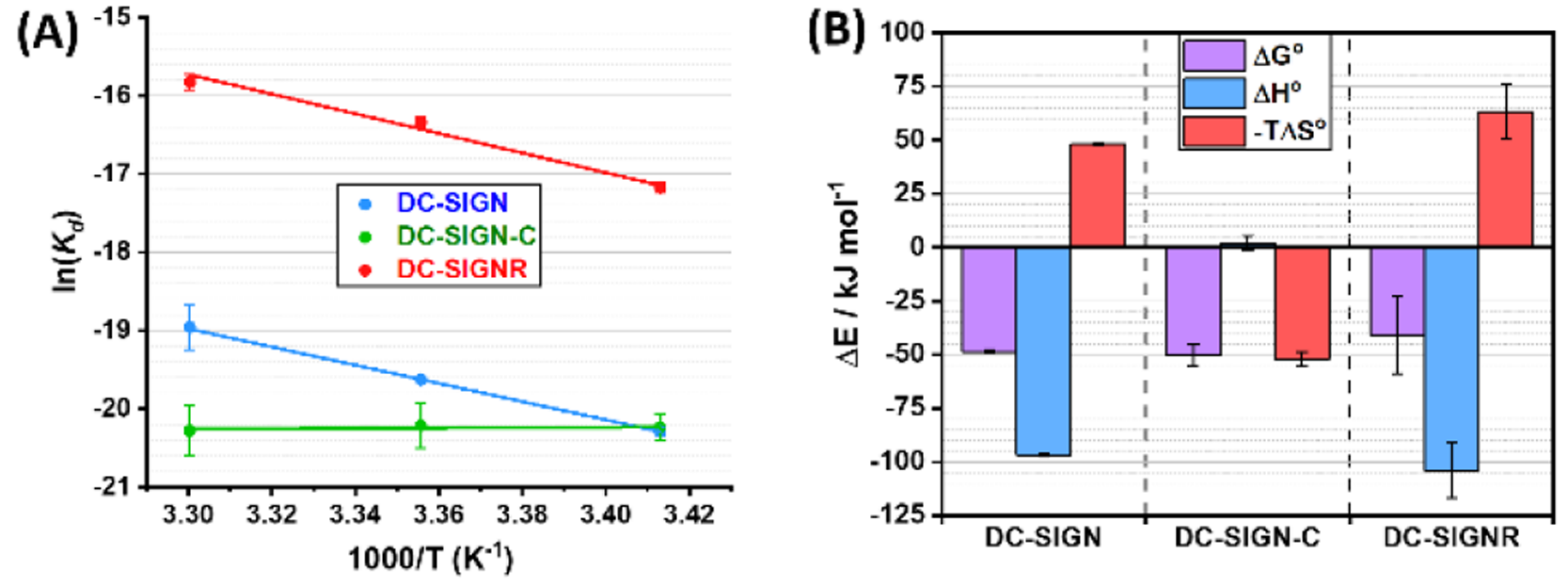
(A) Van’t Hoff analyses of the ln(*K*_d_)–1/*T* relationships for QD-DiMan binding with DC-SIGN (blue), DC-SIGN-C (green), and DC-SIGNR (red). (B) Comparison of the standard (*T* = 298 K) enthalpy (blue), entropy (red), and Gibbs free energy (pink) changes of QD-DiMan binding with DC-SIGN, DC-SIGN-C, and DC-SIGNR. SDs represent fitting errors.

Consistent with our previous results, the binding affinity of DC-SIGN with QD-DiMan is very strong, with an apparent *K*_d_ of ∼1.5 nM at 20 °C, which is >20-fold stronger than that of DC-SIGNR.^20^ To obtain the binding thermodynamics, each binding assay was repeated at three different temperatures (20, 25, and 30 °C; see Figures 2D,E and S11 for detailed fluorescence spectra). Their respective apparent *K*_d_ values were derived from the Hill fits and given in Table 1. Van’t Hoff plots were then constructed to extract the binding enthalpy and entropy changes by combining two Gibbs free energy equations, Δ*G°* = −*RT* ln(*K*_a_) = *RT* ln(*K*_d_) and eq 2, and taking a linear fit of ln(*K*_d_) against the reciprocal of temperature, 1/*T* (eq 3), as shown in Figure 3, where *R* is the gas constant.
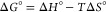
2
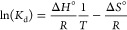
3

The binding thermodynamic parameters for QD-DiMan binding with DC-SIGN/R are summarized in Table 2. On the basis of these data, two conclusions can be drawn. (1) Both DC-SIGN/R bindings with QD-DiMan are enthalpy driven with negative standard binding enthalpy change (Δ*H°*) and entropy change (Δ*S°*) terms. Both Δ*H°* values for DC-SIGN/R binding with QD-DiMan are similar, ∼−100 kJ mol^–1^, which is about 4 times that of monovalent DC-SIGN CRD-DiMan binding measured by ITC (e.g. −25.8 kJ mol^–1^).^50^ Given that glycan binding does not cause conformational changes in CRD,^39,51^ and the QD-OH control without the terminal DiMan shows no measurable binding with DC-SIGN (Figure S9), these results suggest that all four CRDs in both DC-SIGN/R are engaged in glycan binding. This is as expected for enthalpy driven MLGIs. The excellent consistency between the Δ*H* values measured here and that measured from ITC thus confirms our QD-FRET technique is a valid, sensitive new method for investigating the MLGI binding thermodynamics. (2) The multivalent binding Δ*S°* values for DC-SIGN/R-QD-DiMan are ∼5.7 and ∼7 times that of the DC-SIGN CRD-DiMan monovalent binding measured by ITC (−28.5 J mol^–1^ K^–1^),^50^ respectively. Thus, a larger entropic penalty for DC-SIGNR binding with QD-DiMan is responsible for its lower affinity compared to DC-SIGN. The total multivalent binding Δ*S°* consists of changes in translational and rotational entropies of binding partners as well as binding induced entropy changes associated with conformational changes^1^ and solvent molecules. Given that all four CRDs in DC-SIGN/R are engaged in binding and each CRD is most likely to bind a single DiMan molecule,^52,53^ the binding entropy change from the conformation change of monovalent CRD-DiMan binding should be very similar for both lectins. Thus, the higher binding entropic penalty for DC-SIGNR over DC-SIGN is most likely due to a greater reduction of translational and rotational degrees of freedom by forming a smaller number of larger cross-linked protein–QD complexes. The thermodynamic data obtained here are fully consistent with that expected for enthalpy driven MLGIs with different binding modes (i.e., simultaneous binding vs cross-linking).

**Table 2:** Summary of the Binding Thermodynamic Parameters for QD-DiMan Binding with DC-SIGN, DC-SIGN-C, and DC-SIGNR (SDs Represent Fitting Errors)

lectin^a^	Δ**H*°*/kJ mol^–1^	Δ*S*°/J mol^–1^ K^–1^	Δ*G*°/kJ mol^–1^
DC-SIGN	–96.8 ± 0.6	–161 ± 2	–48.6 ± 0.9
DC-SIGN-C	2 ± 4	174 ± 10	–50 ± 5
DC-SIGNR	–100 ± 10	–201 ± 34	–40 ± 20

a ITC measured Δ*H*°, Δ*S*°, and Δ*G*° values for CRD-DiMan monovalent binding are −25.8 kJ mol^–1^, 28.5 J K^–1^ mol^–1^, and −17.3 kJ mol^–1^, respectively.^43^

### Role of C-Terminal Segment in DC-SIGN Multivalent Binding

The CRDs in DC-SIGN/R are linked to the coiled-coil neck domain with some degree of flexibility.^54^ A short C-terminal segment of 16 amino acids length is found at the CRD/neck junction region in DC-SIGN, but it is absent in DC-SIGNR.^38^ Thus, it may act as a steric wedge to maintain the upright CRD orientation and define DC-SIGN’s multivalent binding properties. To probe this, DC-SIGN-C was constructed, labeled with Atto-594, and used for binding studies with QD-DiMan using the same methods as described above.

Interestingly, DC-SIGN-C’s overall QD-DiMan binding profile at 20 °C more closely resembles DC-SIGN than DC-SIGNR. (1) Its binding *K*_d_ is roughly the same as that of DC-SIGN (e.g., 1.6 ± 0.3 vs 1.54 ± 0.07 nM), >20-fold lower (stronger) than that of DC-SIGNR (35 ± 2 nM, see Figure 2C,F and Table 1). (2) Its maximum FRET ratio (*F*_max_) is comparable to that of DC-SIGN and >2-fold that of DC-SIGNR despite the PQR used in the latter being 10 times that of the former (Figure 2F and Table 1). Despite such similarities between DC-SIGN-C and DC-SIGN in QD-DiMan binding at 20 °C, their affinity–temperature dependencies are drastically different. While the *K*_d_ for DC-SIGN-QD-DiMan binding is increased ∼4-fold as the temperature increases from 20 to 30 °C, the *K*_d_ of DC-SIGN-C remains essentially unchanged. Moreover, the maximum FRET ratio for DC-SIGN binding remains almost constant, but that for DC-SIGN-C is reduced considerably with the increasing temperature (Figure 2D,F and Table 1).

The Van’t Hoff plot of DC-SIGN-C-QD-DiMan binding therefore shows little change in the ln(*K*_d_) with changing 1/*T*. The standard binding Δ*H*° and −*T*Δ*S*° terms are obtained as 2 ± 4 and −52 ± 3 kJ mol^–1^, respectively. This thermodynamic profile contrasts greatly with that of DC-SIGN (Δ*H*° = −96.8 ± 0.6 and −*T*Δ*S*° = 48.1 ± 0.6 kJ mol^–1^) or DC-SIGNR (Δ*H*° = −100 ± 10 and −*T*Δ*S*° = 60 ± 10 kJ mol^–1^). Therefore, the removal of the C-terminal segment in DC-SIGN has shifted its MLGI from being enthalpy to entropy driven. Here, the highly favorable binding Δ*H°* observed in DC-SIGN (−97 kJ mol^–1^) is diminished completely in DC-SIGN-C (∼2 kJ mol^–1^). However, the binding is compensated with a strongly favorable standard entropic term (−*T*Δ*S*° = −52 ± 3 kJ mol^–1^), giving rise to almost the same overall binding Δ*G°* (e.g., −49 ± 1 vs −50 ± 5 kJ mol^–1^ for DC-SIGN vs DC-SIGN-C, Table 2 and Figure 3).

**Figure 4:**
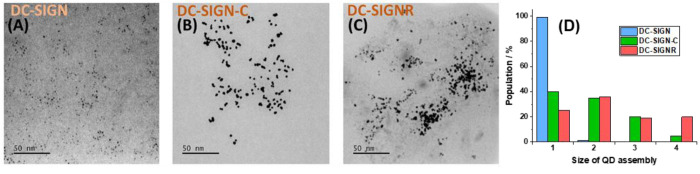
Representative cryo-preserved TEM (contrast inverted HAADF STEM) images of QD-DiMan after binding to (A) DC-SIGN (protein:QD molar ratio, PQR, = 1:1), (B) DC-SIGN-C (PQR = 1:1), or (C) DC-SIGNR (PQR = 10:1). (D) Quantitative analysis of the QD assembly states and cluster sizes after binding to DC-SIGN, DC-SIGN-C, or DC-SIGNR. Note the same PQRs were used here as those used in Figure 2 for the binding affinity quantification.

We further performed “cryo-snapshot S/TEM imaging” to capture the native dispersion states of the lectin–QD complexes in solution to probe lectins’ binding modes.^20^ This was accomplished by rapid plunge freezing of the samples, followed by vacuum drying, and finally loading for S/TEM imaging.^20,55^ The corresponding S/TEM images (Figures 4 and S12) reveal that binding of DC-SIGN gives almost exclusively isolated single QD particles (∼99%), whereas binding of DC-SIGNR results in most of the QDs (∼75%) being clustered, and among those ∼20% are in the group of larger than four particles (Figure 4). This result is fully consistent with our previous observations which also reaffirms the distinct binding modes between DC-SIGN (simultaneous binding with one QD) and DC-SIGNR (cross-linking with multiple QDs).^20^ Interestingly, binding of DC-SIGN-C gives a particle dispersion that is in between those of DC-SIGN and DC-SIGNR: where ∼40% of the QDs are isolated, ∼55% of particles are in groupings of 2 or 3, and only 5% are in groups of >4 particles (Figure 4D). This result shows that the C-terminal segment has made a valid, but not the sole, contribution in maintaining the characteristic tetrameric structure and MLGI properties in DC-SIGN. Its removal results in DC-SIGN-C losing some binding characters of DC-SIGN, but gaining some of DC-SIGNR. This result is also consistent with literature showing that only DC-SIGN, but not DC-SIGNR, expressing cells can bind to the HIV-1 for efficient viral transmission, and removal of the C-terminal segment in DC-SIGN reduces, but not completely demolishes, its HIV-1 binding and transmission ability.^40^

As the C-terminal segment is located at the flexible CRD/neck junction, it may act as a steric barrier to control the CRD flexibility, forcing each CRD to function as an independent unit to retain MLGI specificity. If this is true, then the CRDs in DC-SIGN-C would be less restricted and able to change position/orientation relative to one another more freely than that in DC-SIGN. While this still allows DC-SIGN-C to form simultaneous binding to QD-DiMan to provide high affinity, it would also increase the probability of the CRDs from one DC-SIGN-C molecule to bind to DiMan ligands from different QDs, leading to lectin–QD clustering, which is unlikely to occur in DC-SIGN. This would also lead to CRD–CRD and/or CRD–neck interactions upon QD-DiMan binding, which may account for the observed Δ*H*° penalty. This steric effect of the C-terminal segment can also rationalize the enhancement of Δ*S*° in DC-SIGN-C-QD-DiMan binding, whereby the newly found flexibility of the CRDs would allow for the preservation of the flexibility of both the CRDs and flexible EG_11_ chains of the QD-DiMan scaffold upon binding. Moreover, their binding may even relieve some of the steric strains on the CRDs, leading to the positive binding Δ*S*°. This rationale would also agree with the entropic penalty observed in DC-SIGN, where a more rigid CRD arrangement would cause a loss of the degrees of freedom in the EG_11_ chains upon binding. Thus, the combination of mutagenesis, S/TEM imaging, and QD-FRET analysis is a powerful tool to probe structure–function relationships in MLGIs.

### Investigating MLGI Kinetics

**Figure 5:**
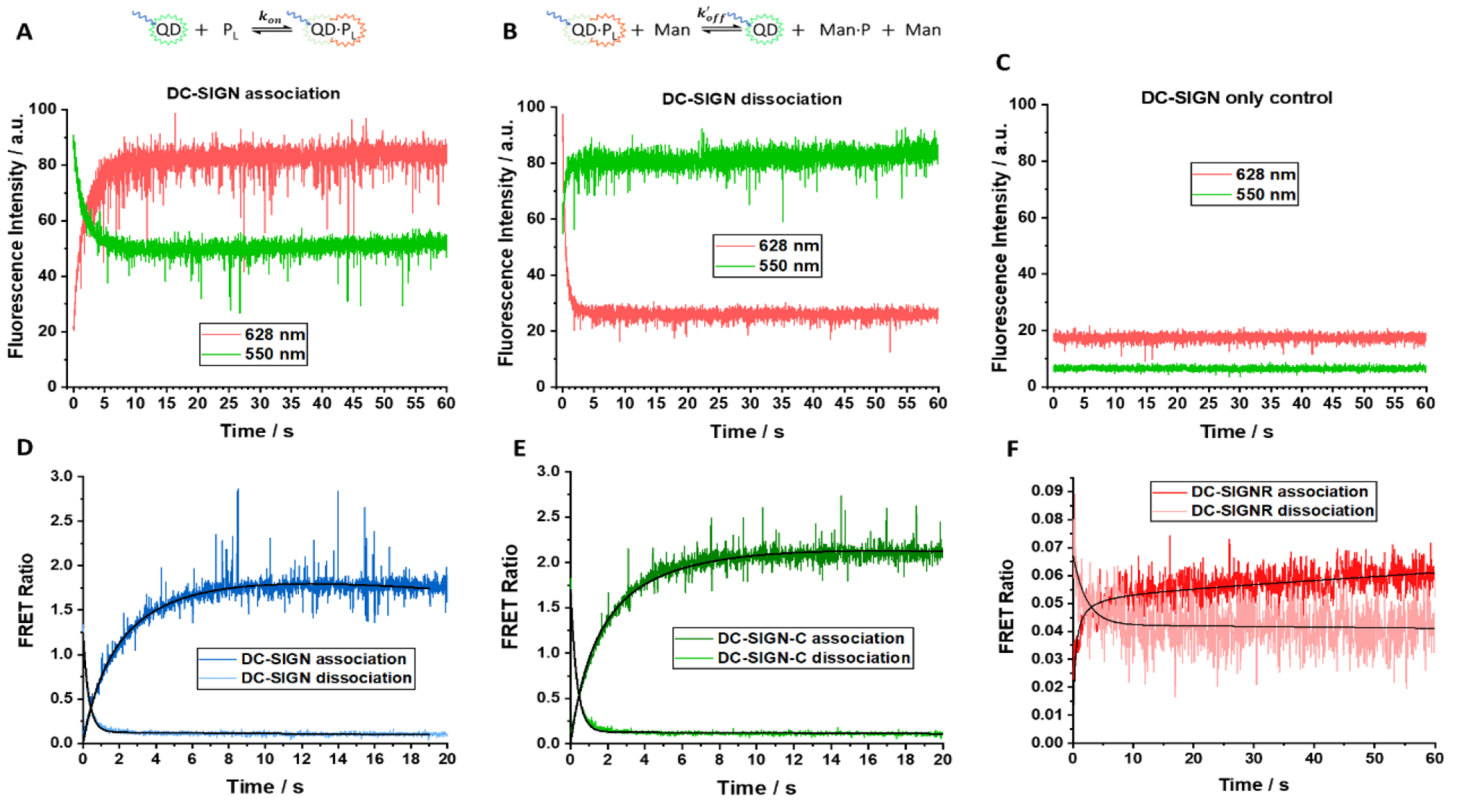
Raw kinetic profile of the fluorescence intensity at 626 nm (red) and 550 nm (green) for the association of (A) QD-DiMan with labeled DC-SIGN, (B) the dissociation of QD-DiMan and labeled DC-SIGN in the presence of excess mannose, and (C) a control containing only labeled DC-SIGN. Kinetic profile of the FRET ratio measured for the association of a 1:1 ratio of QD-DiMan with labeled protein (dark color) and dissociation of bound 1:1 QD-DiMan:protein complex in an excess of mannose (light color) for (D) DC-SIGN, (E) DC-SIGN-C, and (F) DC-SIGNR.

The QD-DiMan–lectin binding kinetics were measured by stopped flow fluorescence via FRET. The association rate was obtained by rapidly mixing QD-DiMan and labeled lectin into an 80 μL cuvette at a 1:1 molar ratio via stopped flow apparatus. Measurements of the QD and dye fluorescence signals were obtained over time (Figure 5A) and were corrected by the dye direct excitation signals (Figure 5C) to provide *I*_D_ and *I*_A_ time profiles, respectively (Figure S13). The FRET ratio was obtained as *I*_A_/*I*_D_, and the averaged FRET ratio–time profiles were fitted by the second-order rate equation to derive the apparent on-rate coefficient, *k*_on_ (eq 4), where *x*_0_ is the initial protein concentration and *a* is a constant to account for the reduction of the QD fluorescence upon transfer from pure water into salt containing binding buffers.^31,36^
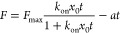
4

Both DC-SIGN and DC-SIGN-C showed very similar association FRET ratio–time profiles for *x*_0_ = 20 nM, which gave maximal FRET ratios similar to those obtained in Figure 2 within 10 s, indicating that saturate binding was achieved (Figure 5D,E). The second-order rate equation fitted nicely for DC-SIGN and DC-SIGN-C, yielding *k*_on_ values of (2.24 ± 0.06) × 10^7^ and (2.92 ± 0.04) × 10^7^ M^–1^ s^–1^ as well as half-lives (*t*_1/2_) of 1.55 ± 0.04 and 1.19 ± 0.02 s, respectively (where *t*_1/2_ = ln(2)/(*x*_0_ + *k*_on_); Table 3). DC-SIGN-C association is slightly faster. A much lower FRET ratio was observed for DC-SIGNR due to its low binding affinity at a 1:1 PQR, which resulted in relatively poor fits due to the low signal-to-noise ratio. This was only slightly improved by taking an average of every five data points, resulting in a time resolution of 0.0625 s. Results showed that despite a relatively rapid initial association (increase of FRET ratio), DC-SIGNR was not able to reach saturation, even after 60 s, and thus the fitting gave a negative *a*-term. Here, the positive *a*-terms (signifying a decrease of FRET ratio over time) observed for DC-SIGN and DC-SIGN-C are likely due to reduction of the QD fluorescence in binding buffer over time. This has been reported previously for other small molecule ligand-capped QDs.^31,36^ The negative *a*-term for DC-SIGNR thus must be the result of another form of association occurring over a much longer timespan, giving rise to an increasing FRET ratio with time.

**Table 3:** Summary Kinetic Parameters for QD-DiMan Binding with DC-SIGN, DC-SIGN-C, and DC-SIGNR (SDs Represent Fitting Errors)

	association rate				dissociation rate				
lectin	*y*_max_	*k*_on_ /×10^7^ M^–1^ s^–1^	*T*_1/2_/s	*a*/×10^–3^ s^–1^	*y*_0_	*y*_eq_	*k*_off_/s^–1^	*t*_1/2_/s	*a*/×10^–3^ s^–1^
DC-SIGN	2.49 ± 0.03	2.24 ± 0.06	1.55 ± 0.04	10.3 ± 0.5	1.261 ± 0.007	0.129 ± 0.001	3.23 ± 0.03	0.215 ± 0.002	1.57 ± 0.09
DC-SIGN-C	2.59 ± 0.01	2.92 ± 0.04	1.19 ± 0.02	5.1 ± 0.2	1.722 ± 0.006	0.131 ± 0.001	2.95 ± 0.02	0.235 ± 0.002	0.93 ± 0.05
DC-SIGNR	0.0532 ± 0.0004	16 ± 1	0.22 ± 0.01	–2.5 ± 0.2	0.067 ± 0.003	0.042 ± 0.001	0.45 ± 0.08	1.5 ± 0.3	0.02 ± 0.01

The kinetic results agree well with that expected for DC-SIGN/R because of their different binding modes. The simultaneous binding DC-SIGN provides a rapid interaction where once initial contact between a CRD and QD-DiMan is formed, it becomes kinetically more favorable for the other CRDs in the same lectin to bind due to the close proximity with the ligand. For DC-SIGNR, it is likely that the initial rapid increase in binding is a result of the simultaneous binding of two CRDs with one QD-DiMan to form a QD-DC-SIGNR intermediate unit. The secondary increase in binding, occurring over a much longer time scale, can be attributed to cross-linking. As cross-linking requires multiple QD-DC-SIGNR intermediate units to interact with each other to form large assemblies, it would be a much slower process. For DC-SIGN-C binding, only minimal amounts of QDs are extensively cross-linked based on the corresponding S/TEM images (∼5%; see Figure 4). Thus, their contributions to the overall FRET signals and binding kinetics may be too small to resolve by our current measurements. Its similar association rate and maximal FRET ratio to those of DC-SIGN suggest that the small assemblies captured by S/TEM imaging are very dynamic, and the CRDs in DC-SIGN-C are more flexible than those in DC-SIGN/R.

Pseudodissociation rates were obtained by injecting a 1:1 premixed QD-DiMan and labeled lectins solution into a binding buffer containing an excess of free d-mannose. A 10^6^ QD molar equivalent of d-mannose was found effective to compete with lectin-QD-DiMan binding (Figure S14), which was used in all dissociation studies. The presence of free mannose greatly reduces the amount of lectins bound to the QD, leading to a decrease of dye FRET signal, a simultaneous recovery of the QD fluorescence, and hence a decrease of the FRET ratio (Figure 5B). A rapid decay in FRET ratio was observed by all three lectins, confirming that QD-DiMan-lectin bindings are specific MLGIs (Figure 5D–F). These FRET decay curves were fitted by a pseudo-first-order rate equation, eq 5 (see the Supporting Information, Section 7.3), as the change in mannose concentration is negligible. Here, *k*_off_^′^ is the apparent pseudo-first-order dissociation rate coefficient.
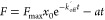
5

As for association, the dissociation rates for DC-SIGN and DC-SIGN-C are similar, with *k*_off_^′^ values of 3.23 ± 0.03 and 2.95 ± 0.02 s^–1^ and half-lives (*t*_1/2_) of 0.213 ± 0.002 and 0.235 ± 0.002 s, respectively (where *t*_1/2_ = ln(2)/*k*_off_^′^). DC-SIGNR appeared to have the slowest rate of dissociation, with *k*_off_^′^ and *t*_1/2_ values of 0.45 ± 0.08 s^–1^ and 1.5 ± 0.3 s, respectively. This is likely due to the difficulty in dissociating the multiple inter- and intra- DC-SIGNR-QD-DiMan interactions within the extensively cross-linked QD-lectin assemblies.

It is worth noting that the *k*_off_^′^ measured in this way is not wholly representative of the true natural dissociation rate, where dissociation and association are in equilibrium and a pair of dissociated binding partners still have chances to rebind. Here, any dissociated protein binding sites will be rapidly occupied by the competitors, making them unable to rebind as that would happen under natural conditions. As a result, the *k*_off_^′^ measured in this way should be faster than natural dissociation. This is apparent by using *k*_off_^′^ to calculate the apparent binding *K*_d_^′^ (via *K*_d_^′^ = *k*_off_^′^/*k*_on_), which would yield values of ∼140 and ∼100 nM for DC-SIGN and DC-SIGN-C, respectively. These values are about 2 orders of magnitude higher than those measured from the thermodynamic FRET assays mentioned above. Therefore, the *k*_off_^′^ derived from competition-based kinetic studies must be treated with caution: it may not reflect the true disassociation rate under natural conditions. Such discrepancies can be quite significant, particularly for multivalent binding systems, where reassociation often occurs under natural conditions due to the close proximity of multiple binding pairs within the binding area.

However, by using the *K*_d_ and *k*_on_ values measured by our QD-FRET thermodynamic (at 20 °C) and kinetic assays, respectively, a more plausible *k*_off_ of ∼0.05 s^–1^ (*k*_off_ = *k*_on_*K*_d_) is obtained for both DC-SIGN and DC-SIGN-C. As the *K*_d_ value was measured under equilibrium conditions, this calculated *k*_off_ should be an accurate reflection of the natural dissociation rate. In fact, this *k*_off_ value broadly agrees with that measured by SPR (e.g., ∼0.1 s^–1^) between surface-immobilized DC-SIGN- and DiMan-coated gold nanoparticles (GNPs, ∼1.2 nm in diameter) without competitors.^56^ Despite some differences in binding environment (surface immobilized vs solution) and core nanoparticle sizes (∼4 vs ∼1.2 nm for QD vs GNP), the good agreement between the calculated *k*_off_ derived from our QD-FRET assays and that measured by SPR for the same pair of lectin and glycan nanoparticles demonstrates that our QD-FRET assays are highly credible for probing a variety of important binding thermodynamics and kinetics for MLGIs.

It is worth noting that the *k*_on_ rate measured by our QD-FRET assay is almost 1000-fold faster than that measured from SPR using surface immobilized DC-SIGN (e.g., ∼10^7^ vs ∼10^4^ M^–1^ s^–1^).^56^ We attribute this difference to different binding environments. As our QD-FRET assays are performed in solution, both binding partners can diffuse freely, greatly increasing the likelihood of collision and thus association. Whereas in SPR, as one binding partner (e.g., DC-SIGN) is immobilized on surface and unable to diffuse, it must rely on the diffusion of the other partner to the surface target sites for any binding to occur. This would result in a significantly slower on rate than that in solution. This is exactly what has been observed here. This result also implies that the binding kinetics measured by surface assays (e.g., SPR and QCM) should not be used to directly predict or explain binding behaviors in solution, and vice versa, due to the influence of binding environments on kinetics. Instead, all binding assays should be performed under the same conditions as those concerned, or at least as close as possible, to obtain meaningful results or explanations. In this regard, the results presented herein have established the glycan-QD FRET assay as a powerful new tool for studying solution phase kinetics and thermodynamics of MLGIs. It is also applicable to other types of binding and biorecognition processes in solution. Although such solution kinetic and thermodynamic data should not be directly used to predict binding interactions on surfaces due to the very different environment, other well-established methods, e.g., SPR and QCM, are well-suited for studying binding interactions on surfaces with one immobilized binding partner.

## Conclusion

In summary, we have significantly expanded the capability of our glycan-QD method in probing MLGIs. Besides providing quantitative binding affinity and binding mode data,^19,20^ we have developed a sensitive QD-FRET technique for the successful dissection of the thermodynamic and kinetic contributions behind affinity enhancing mechanisms in MLGIs with distinct binding modes and for identification of lectin structure–function relationships. We have revealed that the lower QD-DiMan binding affinity for the cross-linking DC-SIGNR, over that of the simultaneous binding DC-SIGN, is a consequence of a larger binding entropy penalty. We have further revealed that the removal of a 16 amino acid C-terminal segment in DC-SIGN, absent in DC-SIGNR, greatly affects its QD-DiMan binding thermodynamic profiles and completely changes the binding from an enthalpy driven into an entropy driven MLGI. These results have allowed us to hypothesize that the entropic gain in removing the C-terminal segment is the result of an increased freedom of the CRDs, which is not present in DC-SIGN naturally. The cryo-S/TEM images of the resulting DC-SIGN-C-QD complex further support the idea that the C-terminal segment may play a key role in maintaining the CRD orientation and therefore in controlling the multivalent specificity of DC-SIGN in binding to multivalent glycans. Together, this work has established the glycan-QDs as a powerful new platform for probing biophysical profiles and structural mechanisms of MLGIs in solution. These data are important for guiding the design of multivalent therapeutics against specific MLGIs, particularly those with unknown structures.

## Experimental Section

### Materials

A CdSe/ZnSe/ZnS core/shell/shell quantum dot (core diameter 3.9 ± 0.5 nm, λ_EM_ = 550 ± 8 nm, quantum yield = 62%) bearing the mixed HDA/TOP/TOPO surface ligands in hexane was purchased from Center for Applied Nanotechnology (CAN) GmbH. H_2_O used was ultrapure (resistance >18.2 MΩ·cm), purified by an ELGA Purelab classic UVF system. All other chemicals and reagents were purchased commercially, and used as received unless stated otherwise.

DHLA-EG_11_-DiMan and DHLA-EG_3_-OH were synthesized in-house using our established protocols.^19,20^ MS: calculated *m*/*z* for C_60_H_111_N_5_O_27_S_2_ (DHLA-EG_11_-DiMan) [M + H]^2+^ 699.84, found 699.92; calculated *m*/*z* for C_32_H_59_N_5_O_9_S_2_ (DHLA-EG_3_-OH) [M + H]^+^ 722.38, found 722.41.

### Preparation of QD-DiMan

QD (53 μM in toluene, 22.5 μL, 1.2 nmol) was precipitated by adding EtOH and centrifuged at 15000*g* for 10 min. The supernatant was discarded, and the pellet was dissolved in CHCl_3_. DHLA-EG_11_-DiMan (2.5 mg, 1.8 μmol) in CHCl_3_, NaOH (0.1 M in EtOH, 2.2 μmol), and MeOH were then added, and the reaction mixture was covered by foil and stirred at rt for 30 min. Hexane was added until the solution became cloudy, and the suspension was centrifuged at 15000*g* for 3 min. The pellet was then dissolved in H_2_O and was washed three times with H_2_O using a 30 kDa MWCO spin filter at 15000*g* for 2 min. All supernatants and washes were collected and combined for QD ligand valency quantification via a sulfur–phenol method.^19,20^ This yielded QD-DiMan with a hydrodynamic diameter (*D*_h_) of 12.4 ± 3.0 nm (mean ±1/2 FWHM (full width at half-maximum)) measured by dynamic light scattering.^20^

### Thermodynamic Studies

All FRET studies were performed in triplicate using a Cary Eclipse fluorescence spectrophotometer using a SUPRASIL quartz cuvette with an optical path length of 1 cm. Samples were excited with λ_ex_ = 450 nm, and the fluorescence spectra were collected from 480 to 750 nm, with intervals (Δλ) of 1 nm. The excitation and emission slit widths and PMT voltages were adjusted to avoid signal saturation at high concentrations. While this would affect the absolute fluorescence signals for both the QD and Atto594, the FRET ratio used in affinity evaluation would be unaffected due to its ratiometric character.^20^ FRET assays were performed by adding protein to QD-DiMan in binding buffer (20 mM HEPES, 100 mM NaCl, 10 mM CaCl_2_, pH 7.8, with 1 mg/mL of BSA to minimize nonspecific interaction and adsorption onto surfaces). All fluorescence spectra were background corrected using the same concentration of lectin only, under identical conditions. Temperature was controlled by a water bath and dry bath for the buffers and samples, respectively. The cuvette temperature was maintained by a built-in temperature control unit using a water pump system.

### Kinetic Studies

Kinetic assays were performed using a TgK Scientific SFA-20 Rapid Kinetics stopped-flow accessory in conjunction with a Cary Eclipse fluorescence spectrophotometer. Measurements were taken using λ_ex_ = 450 nm, and alternating the measurement between the fluorescence intensity at λ_em_ = 550 and 628 nm over time, with a time resolution of 0.0125 s. The apparatus consists of two syringes (A and B) fed to a 80 μL high grade Spectrasil B cuvette via capillary tubes, which then continue to a switch triggered when 0.3 mL of sample is injected. Before measurement, the system was preflushed with H_2_O (40 mL), followed by BSA (1 mg/mL in binding buffer, 2 mL) in syringe A, binding buffer (2 mL) in syringe B, and finally both syringes with binding buffer (10 mL). All kinetics measurements were performed in binding buffer containing 5 μg/mL of His_6_-Cys. We have found previously that addition of His_6_-Cys can enhance the fluorescence and reduce nonspecific interactions for the QD.^19,37^ Associations were obtained by loading syringe A with 2.5 mL of the QD and syringe B with 2.5 mL of protein, both at a final concentration of 40 nM. Dissociations were obtained by loading syringe A with premixed protein and QD, both at a final concentration of 40 nM and syringe B with 2.5 mL of d-mannose (40 mM). Backgrounds were obtained by loading syringe A with binding buffer and syringe B with the protein only (40 nM). For each run, the system was flushed with sample (1.5 mL per syringe) before starting measurements. Each measurement was ran for 60 s before the next injection, where buffer was used to displace the sample once the sample had been completely injected, until the fluorescence signal was observed to drop. Background time profiles were obtained in the same way. Corrected fluorescence profiles were obtained for both association and dissociation experiments by subtracting the background time profiles at the corresponding injection volumes and averaging three measurements at each λ_em_ with consistent fluorescence plateau values. FRET ratio–time profiles were obtained by the ratio of the averaged corrected fluorescence profile at λ_em_ = 628 nm and that at λ_em_ = 550 nm, over time. The kinetic profiles for DC-SIGNR at a 1:1 PQR showed low signal-to-noise due to weak binding; thus, data were smoothed by averaging every five data points, providing a time resolution of 0.0625 s.

### Data Analysis and Fitting

All fluorescence data were analyzed using Microsoft Excel 2016. The FRET ratio data were presented as mean ± standard errors (SEs) of three repeats at each concentration. The FRET ratio–concentration relationships were then plotted and fitted by the Origin software (ver. 2019b) using the relevant equations, taking into account the SEs of each experimental data point, to give the best fits (highest *R*^2^ values). The results from the best fits were then listed in the relevant tables with the standard fitting errors.

## Supporting Information Available

Materials, instruments, and methods showing the production, characterization, and labeling of DC-SIGN, DC-SIGN-C, and DC-SIGNR; preparation and characterization of QD-DiMan hydrodynamic size and surface glycan valency; characterization of QD-DiMan-lectin hydrodynamic sizes, and the kinetic fitting equations (PDF)

## Abbreviations

DC-SIGN: dendritic-cell-specific intercellular adhesion molecule-3-grabbing nonintegrin
DC-SIGN-C: DC-SIGN with the 16 amino acid long C-terminal tail removed
DC-SIGNR: a DC-SIGN related lectin found on endothelial cells
QD: quantum dot
FRET: Förster resonance energy transfer
HRMS: high-resolution mass spectrometry.
