# Supplementary material for: Polyvalent Glycan Quantum Dots as a Multifunctional Tool for Revealing Thermodynamic, Kinetic, and Structural Details of Multivalent Lectin–Glycan Interactions
Source: ACS Appl Mater Interfaces. 2022 Oct 4;14(42):47385–96. doi: 10.1021/acsami.2c11111 (PMC9614721; doi:10.1021/acsami.2c11111)
Supplement: Supplementary file 1 — am2c11111_si_001.pdf [file am2c11111_si_001.pdf]

Supporting Information (SI)

Polyvalent Glycan-Quantum Dots as a Multifunctional Tool for Revealing Thermodynamic, Kinetic and Structural Details of Multivalent Lectin-Glycan Interactions

James Hooper,[1] Yuanyuan Liu,[2,†] Darshita Budhadev,[2] Dario Fernandez Ainaga,[3] Nicole Hondow,[3] Dejian Zhou[2]* and Yuan Guo[1]*

[1] School of Food Science & Nutrition and Astbury Centre for Structural Molecular Biology, University of Leeds, Leeds LS2 9JT, United Kingdom.

[2] School of Chemistry and Astbury Centre for Structural Molecular Biology, University of Leeds, Leeds LS2 9JT, United Kingdom.

[3] School of Chemical and Process Engineering, University of Leeds, Leeds LS2 9JT, United Kingdom.

Corresponding author email: d.zhou@leeds.ac.uk (D.Z.) or y.guo@leeds.ac.uk (Y.G.)

1 Materials, instruments and methods

1.1 Materials

Phenol (>99.5%) was purchased from Alfa Aesar. D-mannose was purchased from Biosynth Carbosynth. Bovine serum albumin (BSA, >99%) was purchased from BioServ UK. A commercial CdSe/ZnSe/ZnS Core/Shell/Shell quantum dot (core diameter: 3.9 ± 0.5 nm, λ_{em} = 550 ± 8 nm, quantum yield = 62%, denoted as QD550) bearing mixed ligands of hexadecylamine, trioctylphosphine and trioctylphosphine oxide in toluene was purchased from Center for Applied Nanotechnology (CAN) GmbH. His_6-Cys peptide was purchased from China peptides. 2-[4-(2-hydroxyethyl)piperazin-1-yl]ethanesulfonic acid (HEPES, >99%); 2-amino-2-(hydroxymethyl)propane-1,3-diol (tris base, >99.8%); calcium chloride ($CaCl_2$, fused granular); chloroform ($CHCl_3$, >99.8%); ethylenediamine tetraacetic acid, disodium salt dehydrate (ETDA, >99%); hydrochloric acid (HCl, ~37%); sodium chloride (NaCl, >99.5%); sodium hydroxide pellets (NaOH, >99%), and sulfuric acid (H_2SO_4, >95%) were purchased from Fischer Scientific. Hexane (>97%) and methanol (MeOH, >99.9%) and were purchased from Sigma-Aldrich. Ethanol absolute (EtOH, >99.97%) was purchased from VWR Chemicals BDH®. Ultra-pure H_2O (resistance >18.2 MΩ.cm) was obtained through an ELGA Purelab classic UVF system.

Common buffers were all made with ultrapure water. These include: loading buffer, 25 mM Tris, 1.25 M NaCl, 25 mM $CaCl_2$, pH 7.8; binding buffer, 20 mM HEPES, 100 mM NaCl, 10 mM $CaCl_2$, pH 7.8; elution buffer, 20 mM HEPES, 100 mM NaCl, 2.5 mM EDTA, pH 7.8; binding buffer for dye labeling, 20 mM HEPES, 100 mM NaCl, 10 mM $CaCl_2$, pH 7.2; and elution buffer for labeling, 20 mM HEPES, 100 mM NaCl, 2.5 mM EDTA, pH 7.2.

1.2 Instruments and Methods

Centrifugation was performed using either a Thermo Scientific Haraeus Fresco 17, Heraeus Multifuge 3SR or a Beckman Coulter Avanti JXN-30 centrifuge, depending on the speed and volume, at room temperature (r.t.) unless otherwise stated. Concentration or washing by centrifugation was carried out using

Sartorius Stedim Lab 30 kDa molecular weight cut-off (MWCO) Vivaspin 500 and Merck Millipore 10 kDa MWCO Amicon Ultra centrifugal filters for QDs and protein, respectively. Dialysis was performed using Thermofischer Scientific 14000 MWCO BioDesign Dialysis Tubing. Evaporation was performed using at reduced pressure using Genevac Concentrator EZ-2 or a Virtis Benchtop K freeze dryer.

High resolution mass spectrometry (HRMS) was performed using a Bruker Daltonics MicroTOF mass spectrometer. Deconvoluted mass values reported are in Da, to four decimal places, and protein labeling efficiency was obtained from the ratio of the integral of the labeled protein HRMS peak to the sum of that of the labelled and unlabeled protein peaks. Ultraviolet-visible light spectrum (UV-vis) was recorded on either a Cary 60 UV-vis spectrophotometer using an Agilent Technologies sub-micro quartz cell with an optical path length of 10 mm or a Thermo Scientific Nanodrop 2000 spectrophotometer with optical path length of 1 mm using a droplet of sample. Hydrodynamic size distribution were obtained using dynamic light scattering (DLS), performed using a Malvern Zetasizer Nano and 10 mm PMMA cuvettes. Mean hydrodynamic size (D_h) values were obtained by fitting the volume percentage hydrodynamic size distribution with a lognormal Gaussian distribution curve.

All FRET studies were performed on a Cary Eclipse Fluorescence Spectrophotometer using a semi-micro quartz cuvette with an optical path length of 1 cm. Samples were excited at a fixed excitation wavelength (λ_{ex}) of 450 nm, corresponding to the minimal absorption of the Atto594 receptor to minimize the direct excitation background, and the fluorescence spectra were collected between emission wavelengths (λ_{em}) of 480 to 750 nm, with intervals ($\Delta\lambda$) of 1 nm. Given the wide range of concentrations used here, the excitation and emission slit widths and PMT voltages were adjusted to ensure the fluorescence signals were not saturated. While the changes of instrument set up will affect the absolute fluorescence signals for both the donor (QD) and acceptor (Atto594), the FRET ratio (I_{dye}/I_{QD}) used to perform affinity quantitation is not affected due to its ratiometric character. This represents a distinct advantage of the ratiometric QD-FRET readout over other assays based on one signal readout: the ratiometric signal is much less sensitive to instrument noises and signal fluctuations, making it highly robust for quantification.[1] All FRET assays were performed in binding buffer (total volume, 400 μL) containing BSA (1 mg/mL) to reduce non-specific adsorption to the walls of the sample tubes and cuvette. The required amounts of protein and QD-glycans were mixed and incubated for 20 min before their fluorescence spectra were recorded. All FRET spectra were corrected by subtracting a background spectrum obtained using the same concentration protein in the BSA containing binding buffer (1 mg/mL). Temperatures were controlled by incubation in a water bath and dry bath for the buffers and samples, respectively. The cuvette temperature was maintained by a Cary single cell Peltier accessory temperature control unit where temperature was sustained using a water pump cooling system.

Kinetic FRET experiments were performed using a TgK scientific SFA-20 Rapid Kinetics stopped-flow accessory in conjunction with the Cary Eclipse Fluorescence Spectrophotometer. Time-dependence measurements were taken using λ_{ex} = 450 nm, and alternating measuring between the fluorescence intensity at 550 nm and 628 nm over time, with a resolution (Δt) of 0.0125 s. The apparatus consists of two syringes (A and B) fed to a 80 μL high grade Spectrasil B cuvette *via* capillary tubes, which then continue to a switch which is triggered when 0.3 mL of sample is injected. Before measurement, the system was pre-flushed with both syringes with H_2O (40 mL), followed by BSA binding buffer (1 mg/ml, 2 mL) in syringe A and binding buffer (2 mL) in syringe B, and finally both syringes with binding buffer (10 mL). All association and disassociation studies and background were recorded in binding buffer containing 5 μg/mL of His_6-Cys. Associations were measured by loading syringe A with 2.5 mL of the QD (40 nM) and syringe B with 2.5 mL of the protein (40 nM). Dissociations were measured by loading syringe A with 2.5 mL of 1:1 mixed QD:protein solution (40 nM each, final concentration, incubated for 20 mins), and syringe B with 2.5 mL of D-mannose (40 mM). Background measurements were obtained by loading

syringe A with 2.5 mL of binding buffer and syringe B with 2.5 mL of the protein (40 nM). For each run, the system was flushed with sample (1.5 mL per syringe) before starting measurements. Each measurement was run for 60 s before the next injection, where buffer was displaced the sample. Corrected fluorescence intensity profiles were obtained for both association and dissociation experiments by subtracting the background time profiles taken at corresponding injection volumes and λ_{em} and averaging 3 measurements at each λ_{em} possessing consistent fluorescence intensity plateau values. FRET ratio time profiles were then obtained by the ratio between the averaged corrected fluorescence profile at λ_{em} = 628 nm and that at λ_{em} = 550 nm, over time. The kinetic profiles for DC-SIGNR at a 1:1 PQR showed low signal to noise ratios due to its weaker binding interaction. Therefore, to improve the signal to noise, the data were smoothed by averaging every five time points, providing a time resolution of 0.0625 s.

All data were analyzed using Microsoft Excel 2016 and graphs were plotted using Origin 2019b software.

2 Synthesis and Characterization of QD-DiMan and QD-EG_3-OH

2.1. Synthesis of ligands. DHLA-EG_{11}-DiMan and DHLA-EG_3-OH ligands were synthesised by copper-free "clicking" interactions between LA-EG_{11}-Cyclooctyne and N_3-EG_2-DiMan or LA-EG_3-Cyclooctyne and N_3-EG_2-OH followed by reduction using TCEP.HCl as reported previously. All the glycans and linkers were synthesised in-house and purified using our previously established protocols.[1,2] MS: calculated *m/z* for $C_{60}H_{111}N_5O_{27}S_2$ (DHLA-EG_{11}-DiMan) $[M+2H]^{2+}$ 699.84, found 699.92; calculated *m/z* for $C_{32}H_{59}N_5O_9S_2$ (DHLA-EG_3-OH) $[M+H]^{+}$ 722.38, found 722.41 (see SI, Figure S1 below).

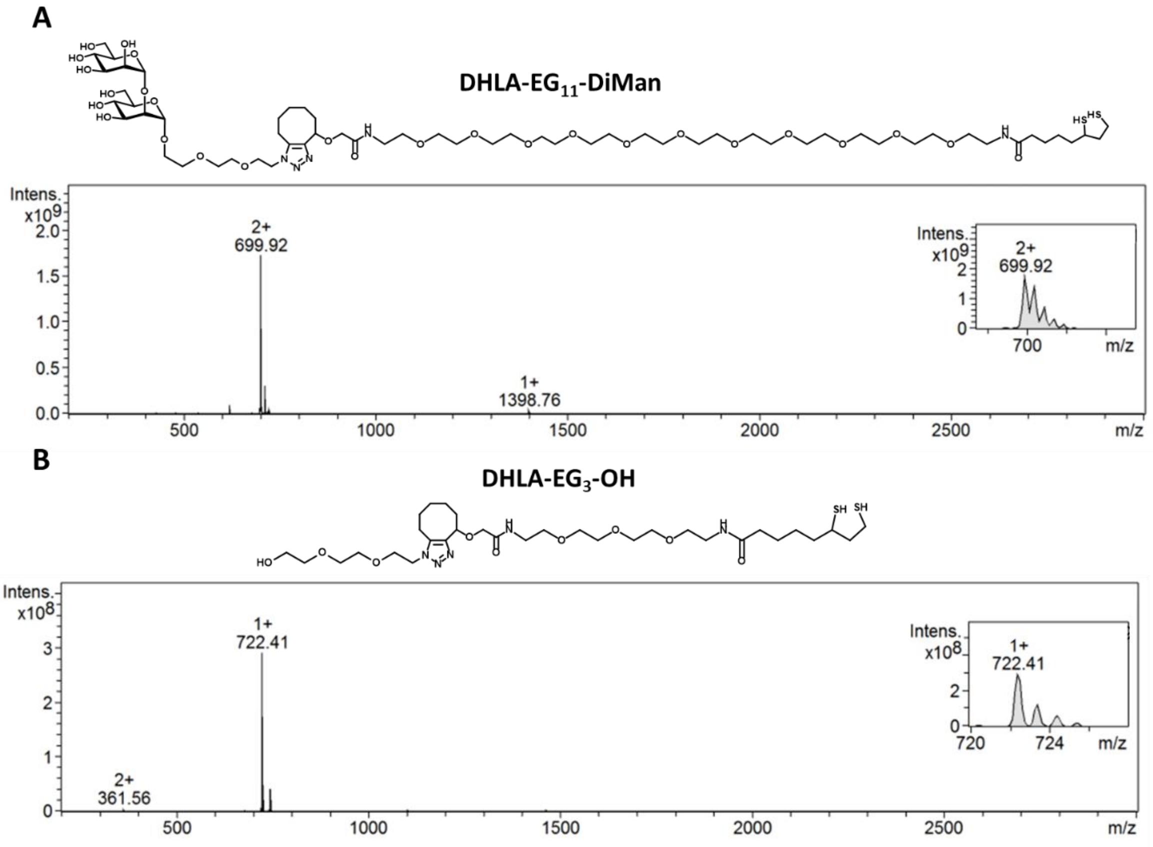

Figure S1. Mass spectrometry data and chemical structures of in-house synthesized ligands for functionalize the QD, (**A**) DHLA-EG_{11}-DiMan and (**B**) DHLA-EG_3-OH.

2.2 preparation and characterization of QD-DiMan/QD-OH.

QD-DiMan:[1,3-5] QD550 (53 µM in toluene, 1.2 nmol) was precipitated by adding EtOH (1.2 ml) followed by centrifugation at 15000×g for 10 mins. The clear supernatant was discarded and the pellet was dissolved in $CHCl_3$. DHLA-EG_{11}-DiMan (2.5 mg, 1.8 µmol) in $CHCl_3$, NaOH (0.1 M in EtOH, 2.2 µmol) and MeOH were then added quickly to the QD solution and the reaction was left to stir, covered, at r.t. for 30 mins. Hexane was then added until precipitation was observed and the suspension was then centrifuged at 15000×g for 3 mins. The clear supernatant was carefully removed and stored for glycan valency evaluation. The QD pellet was dissolved in H_2O, transferred to a 30 kDa MWCO spin filter and then washed 3 times with H_2O, each using a 2 min centrifugation at 15000×g, to remove any unbound free ligands. The supernatant above and the washing solvents were collected and stored for sulfur-phenol quantification of the surface coverage. This yielded a stable QD-DiMan aqueous solution with a mean hydrodynamic size (D_h) of 12.4 ± 3.0 nm (mean ± ½ FWHM, where FWHM = full width at half maximum (see SI, Figure S2).

QD-OH: QD550 (80 µM in toluene, 2 nmol) was precipitated in the presence of EtOH (1.2 ml) and centrifuged at 15000×g for 10 mins. The supernatant was discarded and the pellet was dissolved in $CHCl_3$. DHLA-EG_3-OH (1.0 mg, 1.4 µmol) in $CHCl_3$, NaOH (0.1 M in EtOH, 1.68 µmol) and MeOH were then added quickly and the reaction was left to stir, covered, at r.t. for 30 mins. Hexane was then added until precipitation was observed and the suspension was then centrifuged at 15000×g for 3 mins. The QD pellet was then dissolved in H_2O followed by washing 3 times with H_2O using a 30 kDa MWCO spin filter at 15000×g for 2 mins as above. This yielded a stable QD-OH aqueous solution with a D_h of 9.0 ± 2.9 nm (mean ± ½ FWHM, see SI, Figure S2).

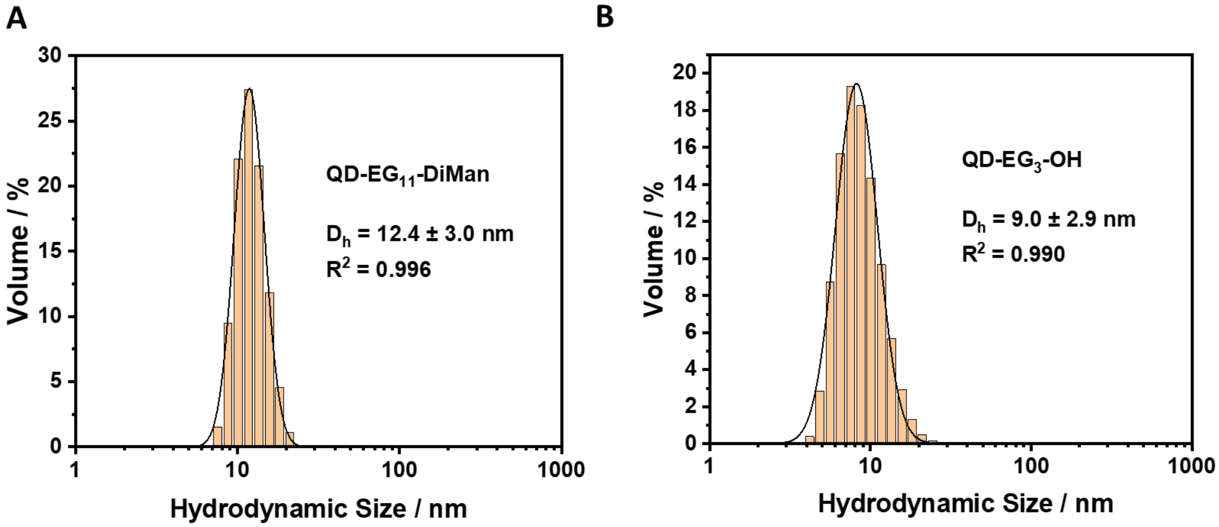

Figure S2. Hydrodynamic size (volume percentage, given as mean ± ½ FWHM, where FWHM is the fitted full width at half maximum) distributions fitted with lognormal Gaussian distribution curves for (**A**) QD-DiMan (QD capped with DHLA-EG_{11}-DiMan ligand, D_h = 12.4 ± 3.0 nm, R^2 = 0.996) and (**B**) QD-OH (QD capped with DHLA-EG_3-OH ligand, D_h = 9.0 ± 2.9 nm, R^2 = 0.990).

2.3. Determination of glycan valency on QD-DiMan.

The glycan ligand valency conjugated to each QD was determined by using a phenol-sulfuric acid method to measure the amount of unbound free ligand.[6] A calibration curve using the N_3-EG_2-DiMan precursor was first constructed. Samples were prepared in duplicates by adding phenol (5 % w/w in H_2O, 80 µL) and sulfuric acid (400 µL) simultaneously to a known concentration of N_3-EG_2-DiMan (in H_2O, 80 µL). After vortex a briefly, the samples were left at r.t. for 30 mins, and their absorbance at 490 nm (A_{490}) were recorded. The resulting A_{490}-concentration relationship was fitted by linear function to yield a calibration curve: Y = (0.0189 ± 0.0003) X μM^{-1} (SI, Figure S3). The supernatants and washing through filtrates, obtained after each QD-DiMan preparation, were combined, freeze dried and dissolved in H_2O. Then phenol-sulfuric acid tests were performed to determine the amount of unbound free ligands. This amount was then subtracted from that used for QD conjugation to give the amount of ligand that have conjugated to the QD. The amount of bound ligand was then divided by the amount of QD-DiMan obtained, giving the number of DHLA-EG_{11}-DiMan ligand bound to each QD as 212 ± 69.

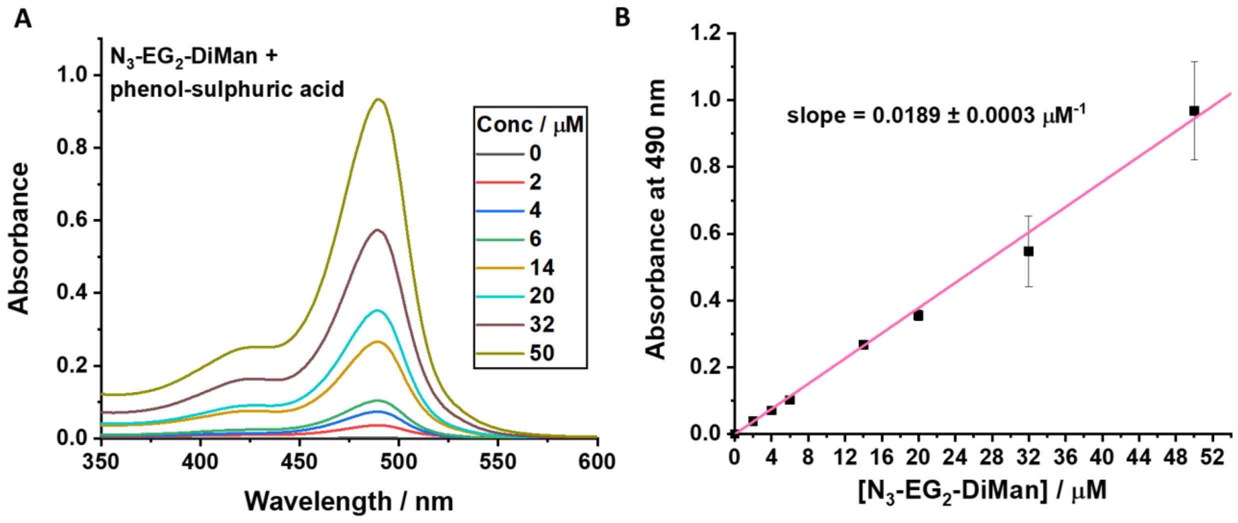

Figure S3. (**A**) The background corrected absorption spectra of varying concentrations of N_3-EG_2-DiMan samples after reaction with 5 % phenol and sulfuric acid (1:5 v/v ratio) and (**B**) a plot of sample absorbance at 490 nm against N_3-EG_2-DiMan concentration fitted by a linear relationship, giving Y = (0.0189 ± 0.0003) X, R^2 = 0.998.

Table S1. Summary of physical parameters derived from the D_h and glycan valency of QD-DiMan. Where, D_h is the hydrodynamic diameter, N is number of ligands per QD, k is the average surface area footprint of each ligand, θ is the average deflection angle, and X is the inter-glycan distance.

Sample	D_h / nm	R^2	N	k / nm²	θ / deg	X / nm
QD-DiMan	12.4 ± 3.0	0.996	212 ± 69	2.3 ± 0.7	16 ± 3	1.7 ± 0.3

The average surface area footprint, k, and average inter-glycan distance of each ligand on the QD surface can be calculated from Eq. (S1-(S3. Where, θ is the average deflection angle of the glycan ligand and r is the average hydrodynamic radius ($r = ½D_h$).[1,7]

$$\mathrm{k} = \frac{4\pi r^2}{N} \tag{S1}$$

$$\theta = \frac{360\sqrt{\frac{k}{\pi}}}{\pi r} = \frac{229.3}{\sqrt{N}} \tag{S2}$$

$$X = 2r \sin\left(\frac{\theta}{2}\right) \tag{S3}$$

2.4 Spectral overlap and Förster radius of QD-Atto594 FRET pair [1,3,9]

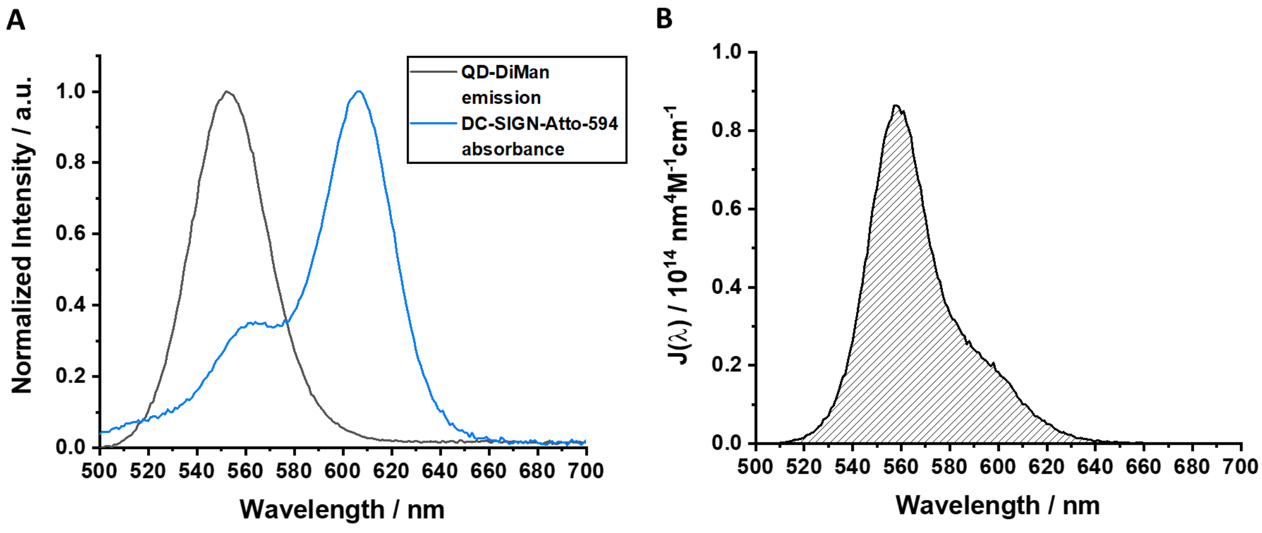

Figure S4. (**A**) Normalized intensity spectra of QD-DiMan fluorescence emission (black) and DC-SIGN-Atto-594 absorption (blue) between 500-700nm; and (**B**) The spectral overlap as a function of wavelength, J(λ), between the QD-EG_{11}-DiMan emission and DC-SIGN-Atto-594 absorption spectra.

The spectral overlap, $J(\lambda)$, was calculated by Eq. S4, where $I_D(\lambda)$ is the normalized donor fluorescence intensity as a function of wavelength and $\varepsilon_A(\lambda)$ is the extinction coefficient of the acceptor as a function of wavelength. The integral of the spectral overlap, the J value, is obtained to be 3.43×10^{15} $nm^4M^{-1}cm^{-1}$.

$$J = \int J(\lambda)\, \mathrm{d}\lambda = \int \frac{I_D(\lambda)\varepsilon_A(\lambda)\lambda^4}{\int I_D(\lambda)\, \mathrm{d}\lambda}\, \mathrm{d}\lambda \tag{S4}$$

Assuming a quantum yield of 50% for QD-DiMan, QY_D, and using these parameters, the Förster radius, R_0, was obtained using Eq. S5, where κ^2 is the dipole orientation factor (κ^2 = 2/3, assuming randomly oriented dipoles) and n_r is the refractive index (n_r = 1.33 for the binding buffer).[5,9] This provided an R_0 value of 57 Å.

$$R_0 = \left(8.79 \times 10^{-5} \cdot \frac{\kappa^2 \cdot QY_D \cdot J}{{n_r}^4}\right)^{\frac{1}{6}} \tag{S5}$$

3 Protein production, labeling and characterization

3.1 Wild type and mutant protein identification

The proteins used were all based off the extracellular domains (ECDs) of DC-SIGN and DC-SIGNR. DC-SIGN and DC-SIGNR plasmids were made in-house and possess their wild type amino acid identities. DC-SIGN Q274C and DC-SIGNR R287C plasmids were made in-house by site-directed mutagenesis, as described in Guo *et al.*, 2016.[8] DC-SIGN-C denotes DC-SIGN with a 16-amino acid deletion at the C-terminal, and its cysteine mutant, DC-SIGN-C Q274C, denotes DC-SIGN Q274C with a 16-amino acid deletion at the C-terminal. The amino acid identities are shown below, whereby asterisks denote residues of similarity between DC-SIGN/-C and DC-SIGNR, and the location of the cysteine mutation is shown in bold.

```
  DC-SIGN: KVPSSISQEQSRQDAIYQNLTQLKAAVGELSEKSKLQEIYQELTQLKAAVGELPEKSKLQEIYQELTRLKAA
DC-SIGN-C: KVPSSISQEQSRQDAIYQNLTQLKAAVGELSEKSKLQEIYQELTQLKAAVGELPEKSKLQEIYQELTRLKAA
 DC-SIGNR: KVPSSLSQEQSEQDAIYQNLTQLKAAVGELSEKSKLQEIYQELTQLKAAVGELPEKSKLQEIYQELTRLKAA
           ***** ***** ************************************************************

  DC-SIGN: VGELPEKSKLQEIYQELTWLKAAVGELPEKSKMQEIYQELTRLKAAVGELPEKSKQQEIYQELTRLKAAVGEL
DC-SIGN-C: VGELPEKSKLQEIYQELTWLKAAVGELPEKSKMQEIYQELTRLKAAVGELPEKSKQQEIYQELTRLKAAVGEL
 DC-SIGNR: VGELPEKSKLQEIYQELTRLKAAVGELPEKSKLQEIYQELTRLKAAVGELPEKSKLQEIYQELTELKAAVGEL
           ******************* ************* ********************* ******** ********

  DC-SIGN: PEKSKQQEIYQELTRLKAAVGELPEKSKQQEIYQELTQLKAAVERLCHPCPWEWTFFQGNCYFMSNSQRNWHD
DC-SIGN-C: PEKSKQQEIYQELTRLKAAVGELPEKSKQQEIYQELTQLKAAVERLCHPCPWEWTFFQGNCYFMSNSQRNWHD
 DC-SIGNR: PEKSKLQEIYQELTQLKAAVGELPDQSKQQQIYQELTDLKTAFERLCRHCPKDWTFFQGNCYFMSNSQRNWHD
           ***** ******** *********  **** ****** ** * ***** **  ********************

  DC-SIGN: SITACKEVGAQLVVIKSAEEQNFLQLQSSRSNRFTWMGLSDLNQEGTWQWVDGSPLLPSFKQYWNRGEPNNVG
DC-SIGN-C: SITACKEVGAQLVVIKSAEEQNFLQLQSSRSNRFTWMGLSDLNQEGTWQWVDGSPLLPSFKQYWNRGEPNNVG
 DC-SIGNR: SVTACQEVRAQLVVIKTAEEQNFLQLQTSRSNRFSWMGLSDLNQEGTWQWVDGSPLSPSFQRYWNSGEPNNSG
           * *** ** ******* ********** ****** ********************* ***  *** ***** *

  DC-SIGN: EEDCAEFSGNGWNDDKCNLAKFWICKKSAASCSRDEEQFLSPAPATPNPPPA
DC-SIGN-C: EEDCAEFSGNGWNDDKCNLAKFWICKKSAASCSRDE
 DC-SIGNR: NEDCAEFSGSGWNDNRCDVDNYWICKKPAA-CFRDE
            ******** ****   *     ***** ** *  **
```

3.2 Production and characterization of DC-SIGN, DC-SIGN-C or DC-SIGNR.

Proteins were expressed from *E. coli.* and purified by sepharose-mannose affinity chromatography as described previously.[1,8]

The concentration of protein was measured by their absorption at 280 nm (A_{280}) *via* UV-vis spectroscopy, where the extinction coefficient at 280 nm for the monomers of DC-SIGN and DC-SIGN-C is 70400 M^{-1} cm^{-1} and for DC-SIGNR is 60890 M^{-1} cm^{-1}.

Correct protein was confirmed by high resolution mass spectrometry (HRMS) (SI, Figure S5) and obtained molecular weight are as following:

DC-SIGN: Calculated molecular weight based on amino acid sequence: 39197.22, found 39202.44

DC-SIGNR: Calculated molecular weight based on amino acid sequence: 37478.99, found 27470.17

DC-SIGN-C: Calculated molecular weight based on amino acid sequence: 37581.41, found 37585.66

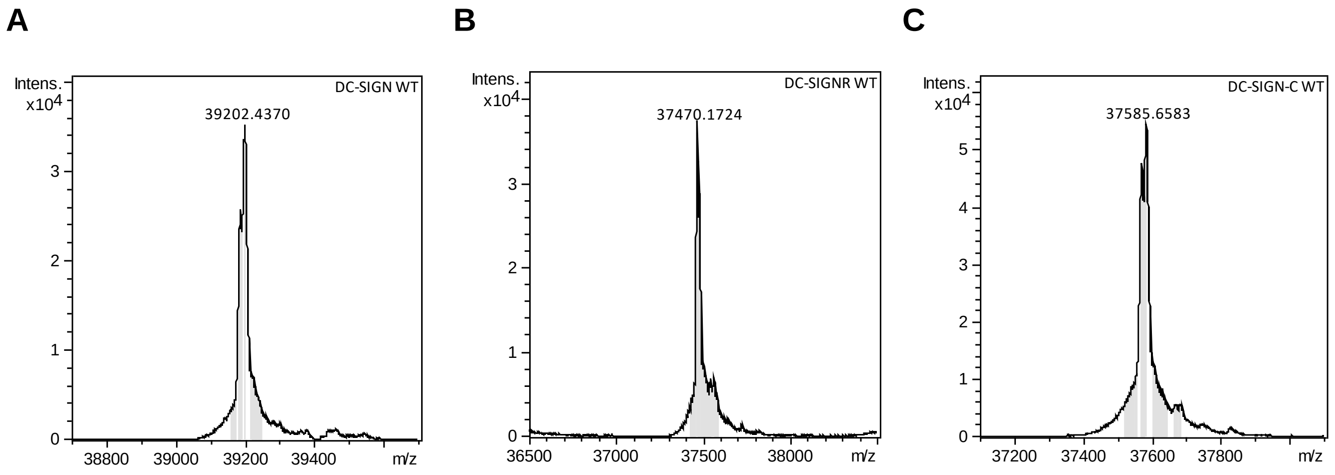

Figure S5. Deconvoluted HRMS spectra of (**A**) DC-SIGN (wild type), (**B**) DC-SIGNR (wild type) and (**C**) DC-SIGN-C (wild type).

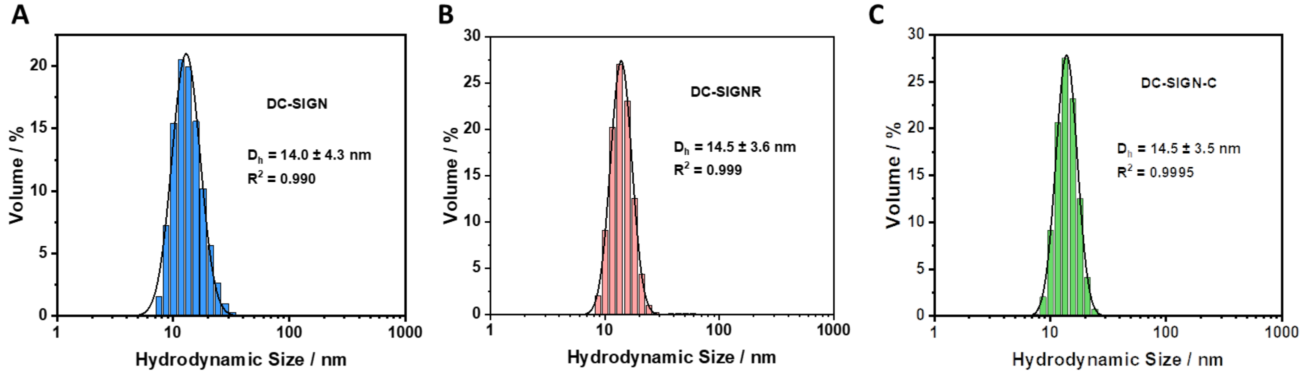

Figure S6. D_h volume distribution histograms fitted with lognormal Gaussian distribution for extracellular segments of wide-type lectins: (**A**) DC-SIGN (D_h = 14.0 ± 4.3, R^2 = 0.990), (**B**) DC-SIGNR (D_h = 14.5 ± 3.6 nm, R^2 = 0.999) and (**C**) DC-SIGN-C (D_h = 14.5 ± 3.5 nm, R^2 = 0.9995). 40 nM proteins were used in DLS studies and their D_h values are given as mean ± ½ FWHM.

3.3 Production and characterization of DC-SIGN Q274C-ATTO594, DC-SIGNR R287C-ATTO594, DC-SIGN-C Q274C-ATTO594 [1,8]

DC-SIGN Q274C, DC-SIGNR R287C or DC-SIGN-C Q274C were produced using the same method as described in Section 3.2.

For protein labeling, the protein was concentrated using 10 kDa MWCO spin filters in buffer containing 20 mM HEPES pH7.2, 150 mM NaCl, 25 mM $CaCl_2$ and then maleimide-ATTO594 (10 mg/mL in dry DMSO) was added at a molar ratio of 1:1.5 for protein monomer: maleimide-ATTO594. The mixture was stirred immediately, covered with aluminium foil and reacted at r.t. for 1.5 hrs with gentle mixing, before

being kept at 4 °C overnight. The mixture was then purified by mannose-sepharose affinity chromatography as described previously.[1,8]

Correct proteins were confirmed by HRMS (SI, Figure S7) and their molecular weights were given below:

DC-SIGN Q274C: calculated protein molecular weight (MW) based on amino acid sequence only: 39172.22, found: 39253.60; and expected MW for [protein + $2Ca^{2+}$]: 39252.40.
DC-SIGN Q274C-ATTO594: calculated molecular weight based on amino acid sequence and dye molecular weight: 40100.22, found 40105.75. Peak area of unlabeled and labeled protein on HRMS was obtained and labelling efficiency was calculated as 87%.

DC-SIGNR R287C: calculated protein molecular weight based on amino acid sequence only: 37425.95; found: 37507.48; and expected MW for [protein + $2Ca^{2+}$]: 37506.10.
DC-SIGNR R287C-ATTO594: calculated molecular weight based on amino acid sequence and dye molecular weight: 38353.95, found 38358.26. Peak area of unlabeled and labeled protein on HRMS was obtained and labeling efficiency was calculated as 85 %.

DC-SIGN-CQ274C: calculated protein molecular weight based on amino acid sequence only: 37556.42; found 37636.62; and expected MW for [protein + $2Ca^{2+}$]: 37636.60.
DC-SIGN-C Q274C-ATTO594: calculated molecular weight based on amino acid sequence and dye molecular weight: 38484.42, found 38490.71. Peak area of unlabeled and labeled protein on HRMS was obtained and labeling efficiency was calculated as 75 %.

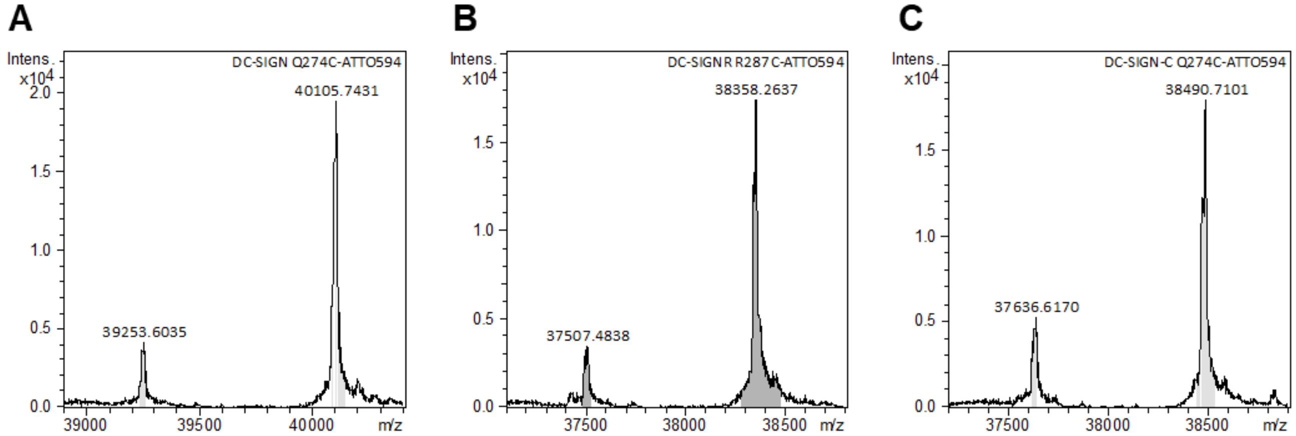

Figure S7. Deconvoluted HRMS spectra of (**A**) DC-SIGN Q274C-ATTO594, (**B**) DC-SIGNR R287C-ATTO594 and (**C**) DC-SIGN-C Q274C-ATTO594.

The concentration of each labeled protein was calculated by using Eq (S6 *via* their UV-vis spectra (SI, Figure S8). Where the protein concentration is denoted as x, the extinction coefficient at 280 nm, ε_{280}, for monomeric DC-SIGN Q274C and DC-SIGN-C Q274C is 70400 M^{-1} cm^{-1} and for monomeric DC-SIGNR R274C is 60890 M^{-1} cm^{-1} and the extinction coefficient at 607 nm, ε_{607}, for ATTO594 is 1.2×10^5 $M^{-1}cm^{-1}$ and its absorption correction factor at 280 nm, CF_{280}, is 0.50.

$$x_{protein} = \frac{A_{280} - CF_{280} \cdot A_{607}}{\varepsilon_{280}} \tag{S6}$$

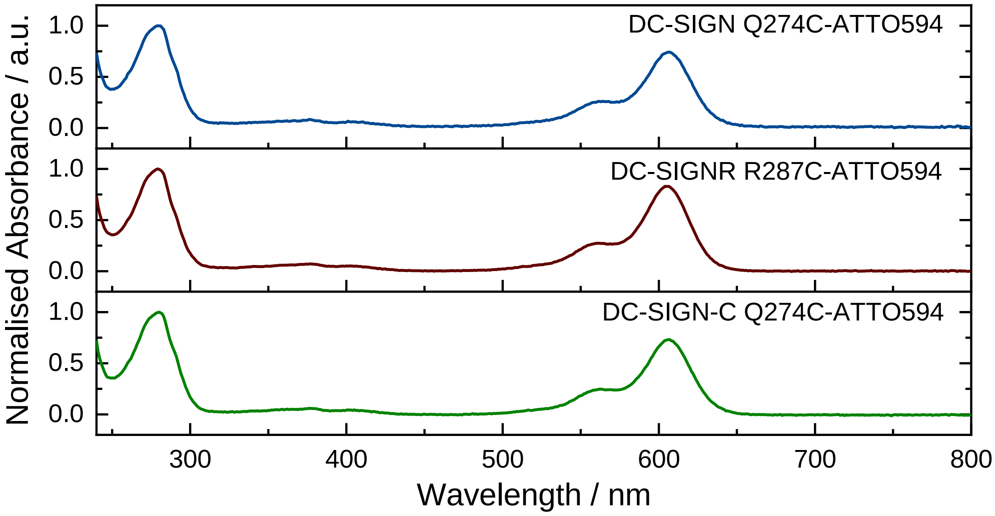

Figure S8. UV-vis spectra of DC-SIGN Q274C-Atto594 (top), DC-SIGNR R287C-Atto594 (center) and DC-SIGN-C Q274C-Atto594 (bottom).

3.4. DC-SIGN Q274C-Atto594 and DC-SIGN-C Q274C-Atto594 binding to QD-OH control

Negligible FRET signal was observed for QD-OH binding with dye labeled proteins, even at high protein: QD-OH ratios (PQRs), confirming that the terminal DiMan is responsible for lectin binding. Therefore, the observed FRET signals are indeed due to specific lectin-glycan interactions.

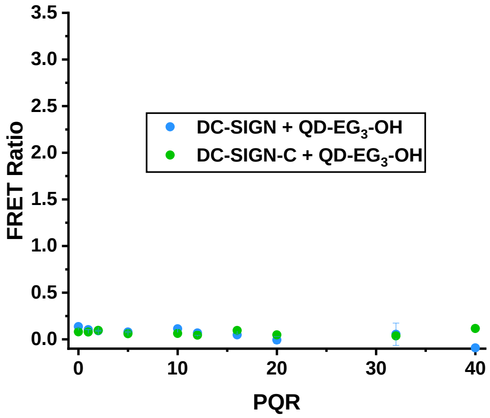

Figure S9. Apparent FRET ratio *vs.* protein: QD ratio (PQR) plots obtained by titrating varying amounts of DC-SIGN (blue) or DC-SIGN-C (green) to a fixed concentration of QD-OH (20 nM). Neither plots showed significant FRET signals, confirming almost no non-specific interactions between the QD-OH control and Atto594 labeled DC-SIGN or DC-SIGN-C.

4 Thermodynamic FRET studies

4.1 QD and protein (Atto-594 labeled) fluorescence-concentration relationships

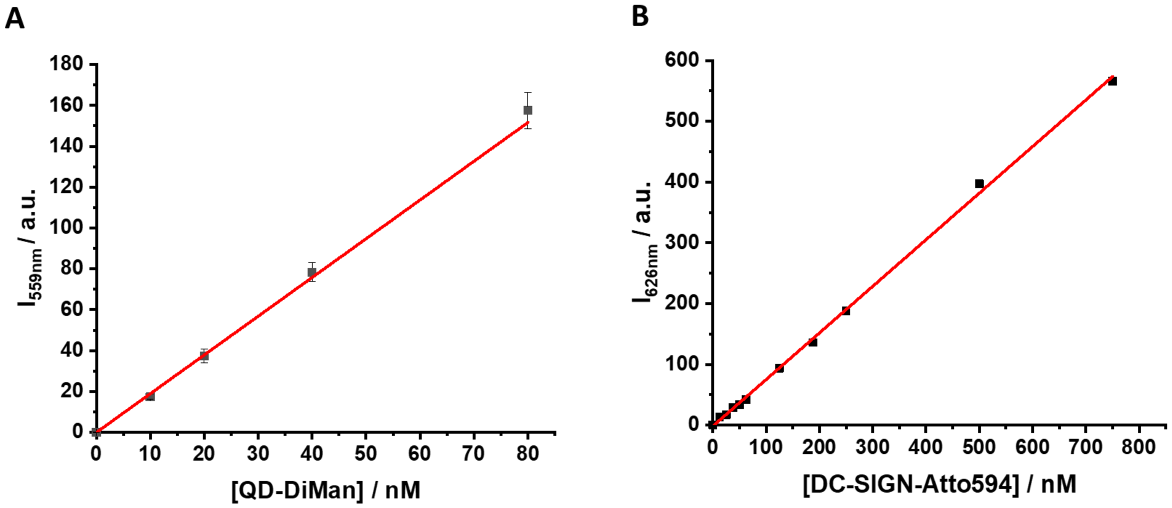

Figure S10. Plots of the fluorescence intensity *vs.* concentration relationships for (**A**) QD-DiMan (λ_{EX} = 450 nm, λ_{EM} = 559 nm; R^2 = 0.997) and (**B**) DC-SIGN (Atto594 labeled, λ_{EX} = 450 nm, λ_{EM} = 626 nm; R^2 = 0.999) fitted by a linear function.

Both the QD and the protein fluorescence increase linearly with concentration across the whole range studied (*e.g.* 0-80 nM for QD-DiMan and 0-800 nM for DC-SIGN-Atto594), confirming no measureable inner filter effect (the presence of significant inner filter effect would cause fluorescence signals to deviate downwardly from linear at high concentrations).

These results also confirm that neither the QD nor the protein (Atto-594 labeled) fluorescence quantum yield is affected by concentration within the range studied.

4.2 Background corrected fluorescence spectra

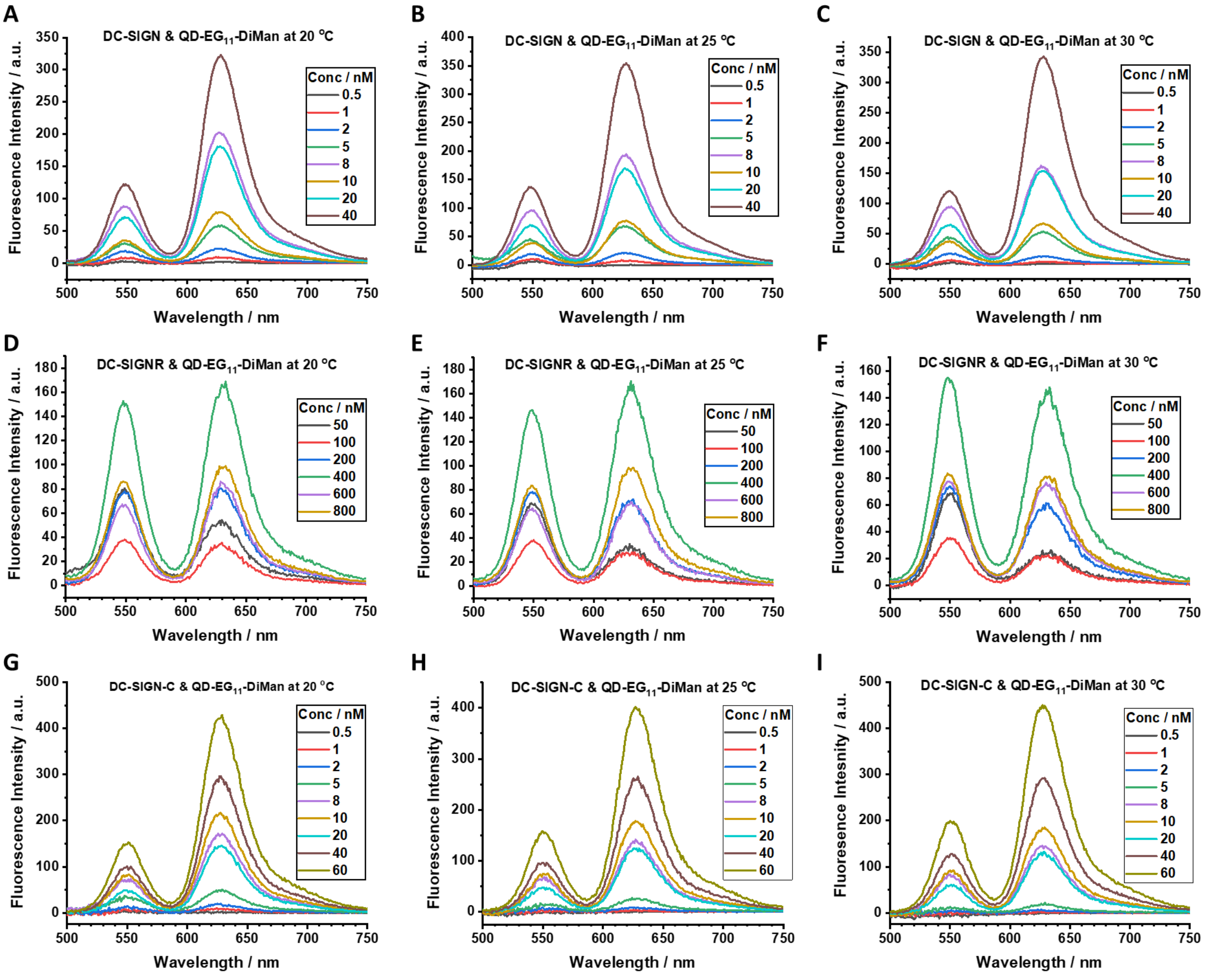

Figure S11. Background corrected fluorescence spectra of the different concentrations of QD-DiMan after mixing with labeled DC-SIGN (PQR = 1:1) at (**A**) 20 °C, (**B**) 25 °C and (**C**) 30 °C; DC-SIGNR (PQR = 10:1) at (**D**) 20 °C, (**E**) 25 °C and (**F**) 30 °C; and DC-SIGN-C (PQR = 1:1) at (**G**) 20 °C, (**H**) 25 °C and (**I**) 30 °C.

5 STEM image

To analyze the dispersion of the QDs after lectin binding, TEM samples were prepared by plunge-freezing into liquid ethane followed by warming under vacuum to capture the QD dispersions in their native dispersed state as demonstrated in our previous papers.[1,10] Briefly, 3.5 μL of the sample suspension was placed onto a plasma-cleaned TEM grid with a continuous carbon support film, blotted, and plunge frozen into liquid ethane. The TEM grids were then warmed to room temperature over several minutes by placing the specimens in a liquid nitrogen cooled storage container in a rotary pumped vacuum desiccator. The samples were plasma cleaned (15 s) and then analyzed using an FEI Titan[3] Themis 300 G2 S/TEM. The samples were imaged using high angle annular dark field scanning transmission electron microscopy (HAADF STEM) mode, which provides atomic number contrast, thereby permitting imaging of the high atomic number quantum dots (brighter) on the low atomic number background (darker). Images presented

in Figure 4 (main manuscript) are contrast inverted, and Figure S12 below shows representative images with the original HAADF contrast.

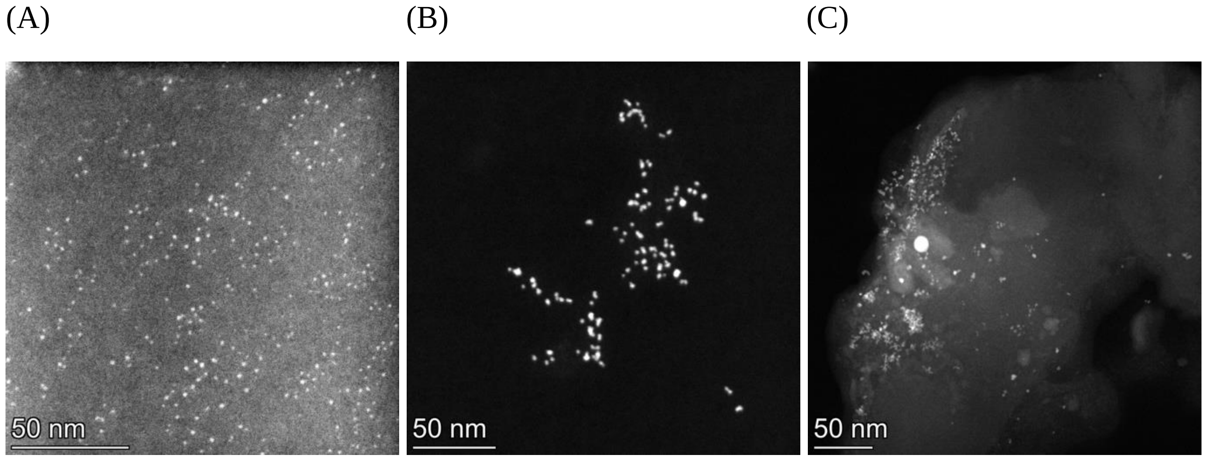

Figure S12. High angle annular dark field scanning transmission electron microscope (HAADF-STEM) images showing the QD-DiMan assemblies after binding with (**A**) DC-SIGN (wild type), (**B**) DC-SIGN-C (wild type) or (**C**) DC-SIGNR (wild type). The QDs are significantly agglomerated upon binding to DC-SIGNR, while those mainly appear as isolated single particles after binding with DC-SIGN, and those after binding with DC-SIGN-C appear to be in between DC-SIGN and DC-SIGNR.

To quantitatively investigate the binding induced QD assembly state, the images presented in Figure 4 were analyzed. Software based approaches to analyze the particles was complicated by the significant overlap of particles in the samples with agglomeration (in particular for DC-SIGNR). Consequently, a manual approach to identify individual or groups of QDs was undertaken in the analysis of the particles present in the images (Figure 4) using the following criteria:

- Particles were considered to be ‘clustered’ if there was no clear boundary between their edges at the magnification and focus of the relevant image. This corresponded to approximately less than 1 nm apart.
- Once a particle had been counted it was marked to avoid the possibility of double counting.

Due to the high contrast difference between the Cd-containing QDs and the carbon support film of the TEM grid images of an appropriate magnification permitted the analysis of between 170 and 400 particles per image.

The analyzed results show that the QD-DC-SIGN particles are predominantly monodispersed, with only 1 % of the population analyzed existing as a pair of QDs at the resolution permitted in the image taken. Analysis of the other two images, taken at the same magnification, do show what initially appear to be larger particles; however, these are often grouping of particles, of 2, 3 or more than 4 particles. This is particularly apparently with the QD-DC-SIGNR sample, where only ~25% of the particles appear to be isolated, and ~20% are in groups of larger than 4 particles, and in many instances, there are over 20 particles in each QD assembly.

6 Kinetic FRET studies

6.1 Corrected FRET time profiles for kinetic studies

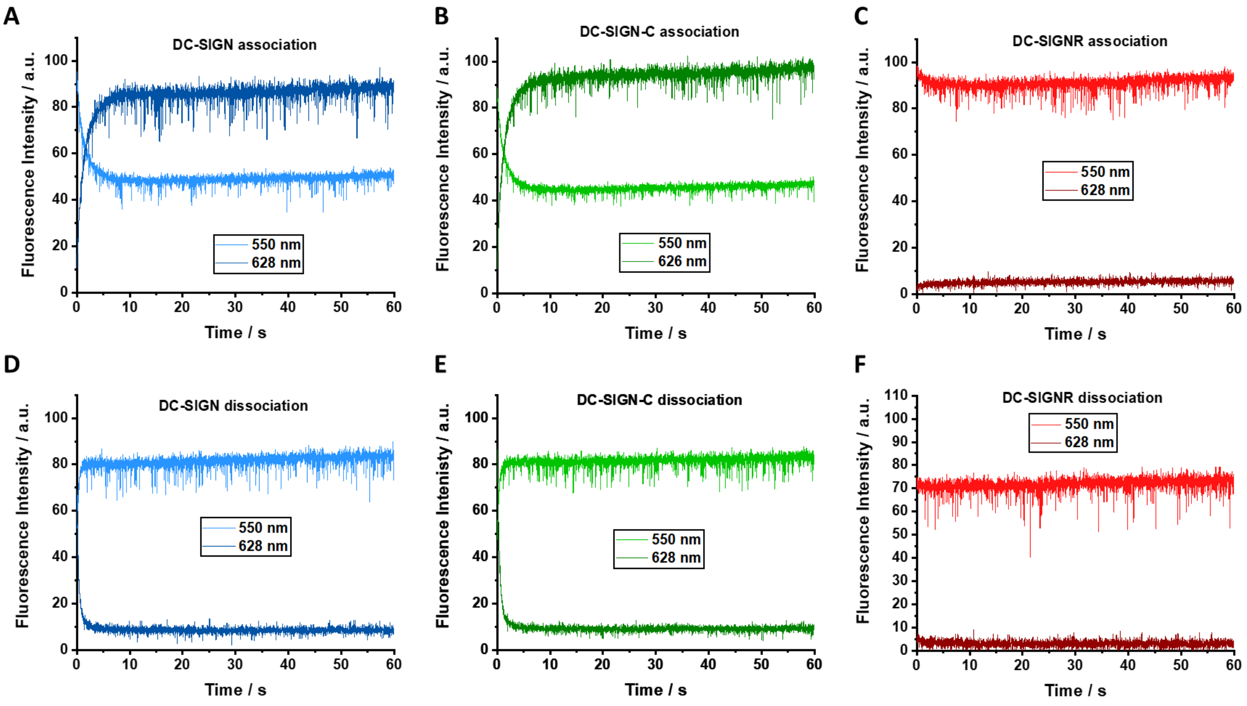

Figure S13. Fluorescence intensity time profiles measuring the emission of 550 nm (lighter color) and 628 nm (darker color) at λ_{EX} = 450 nm for the association of QD-DiMan with (**A**) DC-SIGN, (**B**) DC-SIGN-C and (**C**) DC-SIGNR, and the dissociation of QD-DiMan in the presence of an excess of free mannose with (**D**) DC-SIGN, (**E**) DC-SIGN-C and (**F**) DC-SIGNR.

6.2 Dissociation viability test

A dissociation viability test was performed on the strongest binding protein (DC-SIGN-C) in binding buffer containing 10 µg/mL of His_6-Cys as before to determine the incubation time and mannose concentration required for dissociation of a 1:1 QD-DiMan-lectin complex. Here, DC-SIGN-C and QD-DiMan was mixed at 1:1 molar ratio (both at 44 nM, 360 µL) and incubated at r.t. for 20 mins, and then the fluorescence spectrum was recorded. Then 40 µL D-mannose (400 mM, 1×10^6 eq) was added and fluorescence spectra were recorded every 2 mins. After 20 mins, additional Mannose was added up to 4×10^6 equivalents. Fluorescence spectra were background corrected by their corresponding protein only controls with the same amount of D-mannose. FRET ratios were then obtained and plotted against time or amount of D-mannose.

An incubation time of 2 min and 1×10^6 eq of mannose to the QD concentration were found to be sufficient for maximum dissociation of DC-SIGN-C from QD-DiMan.

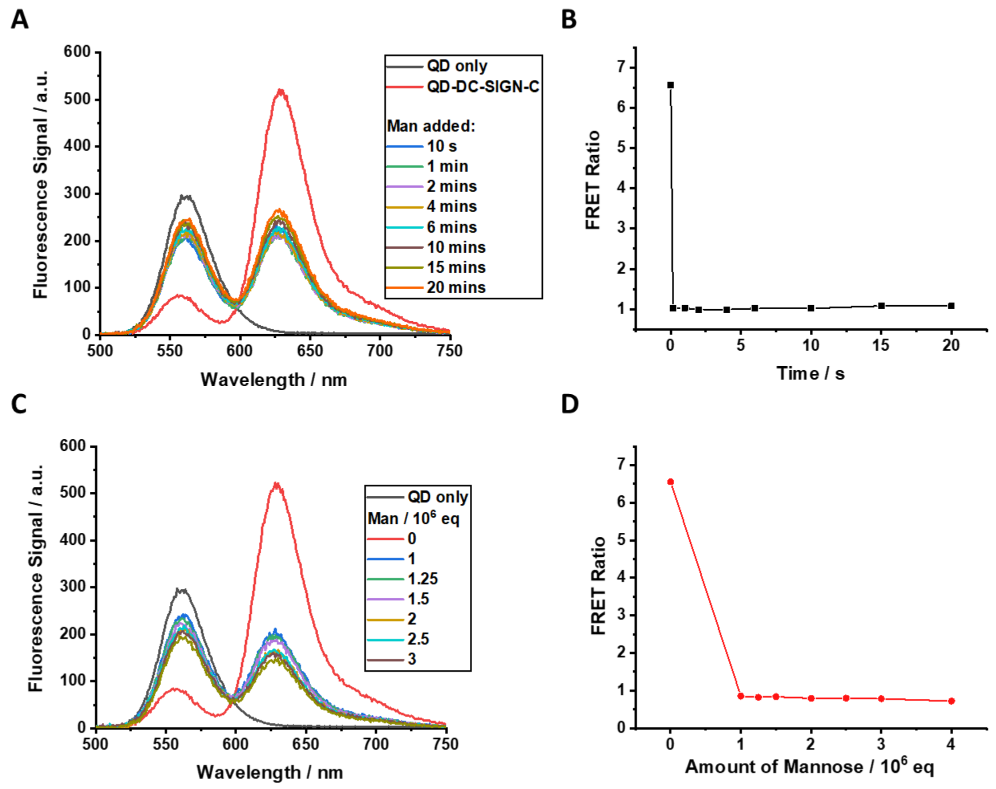

Figure S14. (**A**) Background corrected fluorescence spectra and (**B**) FRET ratio plot of the dissociation of a 1:1 QD-DiMan-DC-SIGN-C complex with 1×10^6 eq of D-mannose (Man) over time; and (**C**) background corrected fluorescence spectra and (**D**) FRET ratio plot of the dissociation and of a 1:1 QD-DiMan-DC-SIGN-C complex with increasing concentrations of Man.

7 Equation derivations

7.1 Thermodynamic equations

The relationship between the binding equilibrium dissociation constant, K_d, and the FRET ratio, F, is derived from the proportional relationship between the FRET ratio and the fraction of bound protein and the Hill equation, as described previously Eq. (S7).[4,8] Where, F_{max} is the maximum FRET ratio, C is the protein concentration and n is the Hill coefficient which denotes the degree of cooperativity between multiple binding species. $n = 1$ is assumed unless otherwise stated, as a 1:1 PQR is used for most measurements which should give no positive or negative cooperativity between individual protein molecules.

$$F = F_{max} \cdot \frac{C^n}{{K_d}^n + C^n} = F_{max} \cdot \frac{1}{1 + (K_d/C)^n} \qquad \text{(S7)}$$

The standard binding enthalpy and entropy changes are obtained by the combination of the Gibbs free energy change terms, Eq. (S8) and Eq. (S9), to give Eq. (S10). Where $K_a = 1/K_d$, is the equilibrium association constant.

Here, a linear fit of $\ln(K_d)$ against $1/T$ can be used to obtain the standard binding enthalpy and entropy changes from the slope and intercept ($\Delta H° = mR$; $\Delta S° = -cR$; where $y = mx + c$).

$$\Delta G° = -RT\ln(K_a) = RT\ln(K_d) \qquad \text{(S8)}$$

$$\Delta G° = \Delta H° - T\Delta S° \quad \text{(S9)}$$

$$\ln(K_d) = \frac{\Delta H°}{RT} - \frac{\Delta S°}{R} \quad \text{(S10)}$$

7.2 Second order FRET rate equation for association

Association kinetics was performed by investigating the interaction between 1 equivalent of protein (P) with 1 equivalent of QD (Eq. (S11) so that a second order rate equation could be used to obtain the association rate coefficient, k_a, where the rate of change of protein concentration, $\frac{d[P]}{dt}$, is directly proportional to the concentration squared, as $[QD] = [P]$ (Eq. (S12). This can then be integrated to provide the relationship between the concentration and time (Eq. (S13).

$$\mathrm{QD} + \mathrm{P} \rightarrow \mathrm{QD} \cdot \mathrm{P} \quad \text{(S11)}$$

$$\frac{d[P]}{dt} = -k_a[QD][P] = -k_a[P]^2 \quad \text{(S12)}$$

$$[P]_t = \frac{[P]_0}{1 + k_a[P]_0 t} \quad \text{(S13)}$$

It can be assumed that the FRET ratio associated with the QD-glycan-protein binding interaction is linearly proportional to the fraction of bound protein (Eq. (S16). As the amount of bound protein is simply the amount of protein that is not free at any time, the concentration of bound protein, $[QD.P]_t$, is equal to $[P]_0 - [P]_t$. Eq. (S14) can then be combined with Eq. (S13) to obtain the relationship between the fraction of bound protein and time, Eq. (S15).

$$\theta_t = \frac{n_{bound\ protein}}{n_{total\ protein}} = \frac{[QD.P]_t}{[P]_t + [QD.P]_t} = \frac{[P]_0 - [P]_t}{[P]_0} \quad \text{(S14)}$$

$$\theta_t = \frac{k_a[P]_0 t}{1 + k_a[P]_0 t} \quad \text{(S15)}$$

The FRET ratio can then be obtained as Eq. (S16) using two assumptions: firstly, that the maximum FRET ratio occurs when all protein has bound, i.e. at $\theta_t = 1$, and secondly, that the FRET ratio decays linearly with time due to a slow decay of QD fluorescence in salt containing binding buffer, where a denotes the linear decay factor.[3] This can then be inserted into Eq. (S15) to provide the relationship between the FRET ratio and time, Eq. (S17), from which the association FRET time profiles are fitted, unless otherwise stated.

$$F_t = F_{max}\theta_t - at \quad \text{(S16)}$$

$$F_t = F_{max}\frac{k_a[P]_0 t}{1 + k_a[P]_0 t} - at \quad \text{(S17)}$$

7.3 Pseudo-first order FRET rate equation for dissociation

Dissociation kinetics were performed using an excess of free D-mannose (Man) to readily compete with the 1:1 QD-glycan-protein binding interaction, Eq. (S18). Due to this, a pseudo-first rate order can be derived to obtain the pseudo-dissociation rate coefficient, k'_d, where the rate of change of the concentration of bound protein, $\frac{d[QD.P]}{dt}$, is linearly proportional to this concentration due to the negligible change in the concentration of mannose, Eq. (S19). This can be integrated to obtain the relationship between complex concentration and time, Eq. (S20).

$$QD.P + Man \rightarrow QD + P + Man \quad \text{(S18)}$$

$$\frac{d[QD.P]}{dt} = -k[QD.P][Man] = -k'_d[QD.P] \quad \text{(S19)}$$

$$[QD.P]_t = [QD.P]_0 e^{-k'_d t} \quad \text{(S20)}$$

The change in FRET ratio over time, Eq. (S22), can then be derived using Eq. (S14) and Eq. (S16) to obtain the relationship between the FRET ratio and the concentration of bound protein, Eq. (S21), where $F_0 = F_{max}$ and the protein concentration is equal to $[QD.P]_0 - [QD.P]_t$, which is inserted into Eq. (S20). Then Eq. (S22) is used to fit all dissociation FRET-time profiles, unless otherwise state.

$$F_t = F_0\,\theta_t - at = F_0 \cdot \frac{[QD.P]_t}{[P]_t + [QD.P]_t} - at = F_0 \frac{[QD.P]_t}{[QD.P]_0} - at \quad \text{(S21)}$$

$$F_t = F_0 e^{-k'_d t} - at \quad \text{(S22)}$$

References

(1) Guo, Y.; Nehlmeier, I.; Poole, E.; Sakonsinsiri, C.; Hondow, N.; Brown, A.; Li, Q.; Li, S.; Whitworth, J.; Li, Z.; Yu, A.; Brydson, R.; Turnbull, W. B.; Pohlmann, S.; Zhou, D. Dissecting Multivalent Lectin-Carbohydrate Recognition Using Polyvalent Multifunctional Glycan-Quantum Dots. *J. Am. Chem. Soc.* **2017,** *139*, 11833-11844.

(2) Budhadev, D.; Poole, E.; Nehlmeier, I.; Liu, Y.; Hooper, J.; Kalverda, E.; Akshath, U. S.; Hondow, N.; Turnbull, W. B.; Pöhlmann, S.; Guo, Y.; Zhou, D. Glycan-Gold Nanoparticles as Multifunctional Probes for Multivalent Lectin-Carbohydrate Binding: Implications for Blocking Virus Infection and Nanoparticle Assembly. *J. Am. Chem. Soc.* **2020,** *142*, 18022-18034.

(3) Zhou, D.; Ying, L.; Hong, X.; Hall, E. A.; Abell, C.; Klenerman, D. A Compact Functional Quantum Dot-DNA Conjugate: Preparation, Hybridization, and Specific Label-Free DNA Detection. *Langmuir* **2008,** *24*, 1659-1664.

(4) Zhang, Y.; Zhang, H.; Hollins, J.; Webb, M. E.; Zhou, D. Small-Molecule Ligands Strongly Affect the Förster Resonance Energy Transfer Between A Quantum Dot and A Fluorescent Protein. *Phys. Chem. Chem. Phys.* 2011, **13**, 19427-19436.

(5) Wang, W.; Guo, Y.; Tiede, C.; Chen, S.; Kopytynski, M.; Kong, Y.; Kulak, A.; Tomlinson, D.; Chen, R.; McPherson, M.; Zhou, D. Ultraefficient Cap-Exchange Protocol To Compact Biofunctional Quantum Dots for Sensitive Ratiometric Biosensing and Cell Imaging. *ACS Appl. Mater. Interfaces* **2017,** *9*, 15232-15244.

(6) Saha, S. K.; Brewer, C. F. Determination of the Concentrations of Oligosaccharides, Complex Type Carbohydrates, and Glycoproteins Using the Phenol-Sulfuric Acid Method. *Carbohydrate Res*. 1994, **254**, 157-167.

(7) Hill, H. D.; Millstone, J. E.; Banholzer, M. J.; Mirkin, C. A. The Role Radius of Curvature Plays in Thiolated Oligonucleotide Loading on Gold Nanoparticles. *ACS Nano* 2009, **3**, 418-424.

(8) Guo, Y.; Sakonsinsiri, C.; Nehlmeier, I.; Fascione, M. A.; Zhang, H.; Wang, W.; Pohlmann, S.; Turnbull, W. B.; Zhou, D. Compact, Polyvalent Mannose Quantum Dots as Sensitive, Ratiometric FRET Probes for Multivalent Protein-Ligand Interactions. *Angew. Chem. Int. Ed.* **2016,** *55*, 4738-4742.

(9) Charbonnière, L.J. and Hildebrandt, N. Lanthanide Complexes and Quantum Dots: A Bright Wedding for Resonance Energy Transfer. *Eur. J. Inorg. Chem*. 2008, **2008**, 3241-3251.

(10) Hondow, I.; Ilett, M.; Wills, J.; Micklethwaite, s.; Brydson, R.; Brown, A. Quantifying the Dispersion of Nanoparticles by Electron Microscopy. *J. Nanopart. Res.* **2012,** *14*, art no. 977.
